# Protocol for the conceptualization and evaluation of a screening-tool for fitness-to-drive assessment in older people with cognitive impairment

**DOI:** 10.1371/journal.pone.0256262

**Published:** 2021-09-01

**Authors:** Leonhard Zellner, Florian Herpich, David Brieber, Margit Herle, Peter Zwanzger, Alexander Brunnauer

**Affiliations:** 1 kbo-Inn-Salzach-Klinikum, Clinical Center for Psychiatry, Psychotherapy, Psychosomatic Medicine, Geriatrics and Neurology, Wasserburg/Inn, Germany; 2 Schuhfried GmbH, Mödling, Austria; 3 Department of Psychiatry and Psychotherapy, Ludwig-Maximilians University Munich, Munich, Germany; Cardiff University, UNITED KINGDOM

## Abstract

**Introduction:**

Due to aging and health status people may be subjected to a decrease of cognitive ability and subsequently also a decline of driving safety. On the other hand there is a lack of valid and economically applicable instruments to assess driving performance.

**Objective:**

The study is designed to develop a valid screening-tool for fitness-to-drive assessment in older people with cognitive impairment externally validated on the basis of on-road driving performance.

**Methods:**

In a single-centre, non-randomized cross-sectional trial cognitive functioning and on-road-driving-behavior of older drivers will be assessed. Forty participants with cognitive impairment of different etiology and 40 healthy controls will undergo an extensive neuropsychological assessment. Additionally, an on-road driving assessment for external validation of fitness to drive will be carried out. Primary outcome measures will be performance in attention, executive functions and visuospatial tasks that will be validated with respect to performance on the on-road-driving-test. Secondary outcome measures will be sociodemographic, clinical- and driving characteristics to systematically examine their influence on the prediction of driving behavior.

**Discussion:**

In clinical practice counselling patients with respect to driving safety is of great relevance. Thus, having valid, reliable, time economical and easily interpretable screening-tools on hand to counsel patients is of great relevance for practitioners.

**Ethics and dissemination:**

Ethics approval was obtained from the Ethics Committee at the Ludwig-Maximilians-University Munich. The trial results will be disseminated through peer-reviewed publications and various conferences.

**Trial registration:**

18–640.

Trial registration: German Clinical Trials Register.

Registration number: DRKS00023549.

## 1 Introduction

Mobility is important for daily life functioning and there is evidence that driving cessation, e.g. in cases of aging or chronic illness, affects social and economic well-being with impact on health functioning [1]. About 70% of patients with a psychiatric disease–organic mental disorders included–possess a driver license and 77% of them are using a motor vehicle on a regular basis [2]. A variety of psychiatric and neurological diseases as well as medical conditions are associated with neurocognitive impairments that are closely linked with driving performance [3–7]. It could be demonstrated that progressing deterioration of the above-mentioned cognitive functions leads to a decline of driving ability and increases the risk of driving errors and accident frequency [8–11].

In clinical practice counselling patients with respect to driving ability is often accompanied by uncertainties both on the clinician’s and patient’s side [10, 12]. Screening-tools like the MMSE (Mini-Mental-State-Examination), which allows an assessment of the cognitive status of a person, are often used in clinical practice, even though their predictive value with respect to driving performance is unclear [13]. Results of neuropsychological tests, especially in domains like attention, executive functions and visuospatial abilities seem to be promising predictors of driving performance [14–16].

Hird et al. [14, 17] outlined the high prognostic validity of the Trail-Making-Test (TMT) and its two subtests A (attention respectively processing speed) and B (executive function respectively cognitive flexibility) as a predictor for driving behavior in dementia, mild cognitive impairment and several other neurological diseases. Correlations with driving performance ranging from medium to high for the TMT-A and TMT-B could be demonstrated [18–24]. Furthermore, visuospatial abilities, since they are essential in performing a vast variety of driving relevant tasks such as positioning and distance estimation are an important prerequisite for safe driving [15]. On an individual test level, the Judgement-of-Line-Orientation-Test (JLO) has been suggested as a valuable predictor with medium correlations regarding driving performance [1, 25–28].

Neuropsychological assessment may therefore provide a practical off-road window into the functional status of cognitive domains relevant for driving [1, 29]. Besides, studies consistently revealed that especially composite batteries. are the best predictor concerning driving performance [29–31]. They comprise combinations of tests and multiple neuropsychological tools, which assess different driving relevant cognitive abilities, However, it is important to emphasize that the criterion related validity of these test combinations is not sufficiently clarified [32].

The purpose of this study is the evaluation of a computerized test-set for a fitness-to-drive assessment in older patients with cognitive impairments of different etiology. We hypothesize that cognitively impaired subjects should differ significantly from a healthy comparison group in an on-road driving test. Furthermore, we expect especially visuospatial abilities, attentional and executive functions to predict driving performance best. The screening tool should differentiate between safe and unsafe drivers with high diagnostic accuracy, estimated at more than 80%, and therefore exceeding individual neuropsychological tests.

## 2 Methods and analysis

### 2.1 Study design

The study is designed as a single-centre, non-randomized cross-sectional-study at the kbo-Inn-Salzach-Klinikum Wasserburg/Inn, Department of Neuropsychology. We expect the recruitment period to last about 24 months.

### 2.2 Recruitment

Forty psychiatric inpatients with cognitive impairment of different etiology will be recruited from therapeutic wards of the hospital. An examination of the patient’s mental and physical condition as well as the medication status will be issued by the treating physicians. The control group will comprise 40 healthy participants recruited from the hospital staff and by advertisement via information-flyers. Control participants will also be screened with respect to medications and health status by professional clinicians. Before examination, full information of the study design will be provided. Before enrollment participants will provide written informed consent. The control group will be financially reimbursed with 25 euros to reimburse for travelling costs.

Eligibility criteria:

The following general inclusion criteria are defined:

- Age ≥ 50 years
- Valid driver license
- Regular participation in road traffic (more than 3000 km/year at the time of the study)
- Good German language skills
- MMSE ≥18 and <27 for the cognitively impaired patients
  ○ 20 patients with organic, including symptomatic disorders (F00-F09)
  ○ 10 with affective disorders (F30-F39)
  ○ 10 with schizophrenia, schizotypal or delusional disorders (F20-F29) or neurotic, stress-related and somatoform disorders (F40-F48)
- MMSE ≥ 27 for the healthy controls

The following general exclusion criteria are defined:

- Moderate to severe dementia (MMSE < 18)
- Severe psychiatric, neurological or medical disease
- Current radio- or chemotherapy
- Visual impairment (visual acuity worse than 60%; visual field <140˚, double vision, hemi-neglect)

### 2.3 Experimental schedule

**Fig 1:**
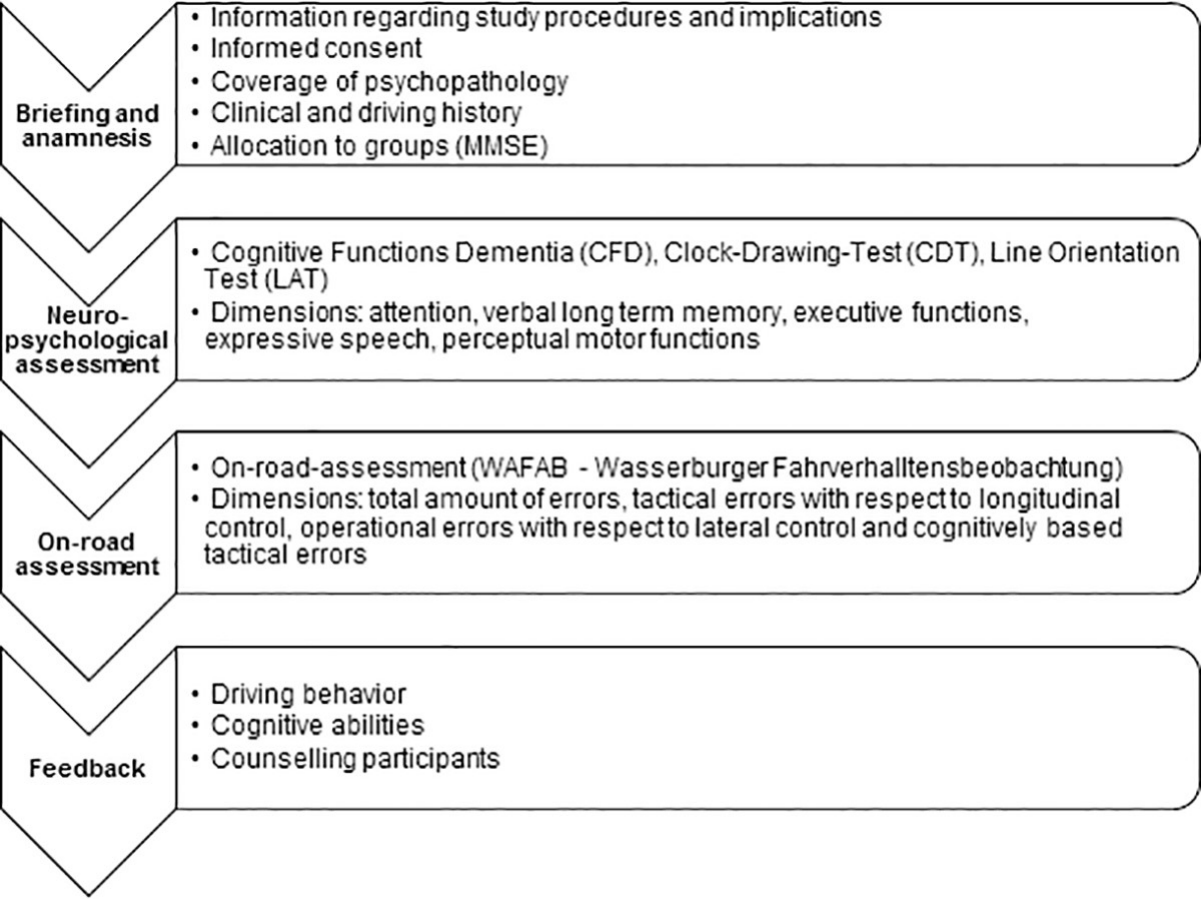
Flowchart of the study procedure.

All data, including demographics and cognitive measures will be collected during one visit, in the midmorning for the purpose of standardization. At first participants will be informed about the content and implementations of the study, followed by written informed consent. Subsequently clinical and driving history will be collected systematically. The participants will be assigned to experimental or control group when recruited. Following this, the participants will undergo a comprehensive neuropsychological examination, which comprises the test battery “Cognitive Functions Dementia” (CFD) [33], the “Line Orientation Test” (LAT; abbreviation based on the German test name) [34] and the “Clock-Drawing-Test” (CDT) [35]. The duration of the assessment will be about 120 minutes. After a short rest period we will proceed with the 50 minutes on-road assessment. Finally, the participants will be informed about the results of the neuropsychological and on-road assessment and counselled with respect to driving safety. A flowchart of the study procedure is given in Fig 1.

### 2.4 Assessment instruments

#### 2.4.1 Clinical interviews

In order to assess sociodemographic (e.g. participant’s age, gender, civil status, years of education), clinical (psychiatric, neurologicaland medical diseases, psychiatric inpatient stays, visual impairments, psychopharmacological treatment) as well as driving related data (years of possessing a driver license, use of automobile, driving abstentions, fines, crashes, limited mobility) we will use standardized questionnaires and interviews.

#### 2.4.2 Rating scales

Mini-Mental-State-Examination (MMSE) [36], Mini-Symptom-Checklist (Mini-SCL) [37], Perceived Deficits Questionnaire (PDQ) [38].

#### 2.4.3 Neuropsychological assessment

As part of the computerised neuropsychological assessment the test set CFD [33] covers neurocognitive functions relevant for neurocognitive disorders according to DSM-5 such as attention, verbal long term memory, executive functions, expressive speech and perceptual motor functions and includes for example the tests TMT-L (Trail-Making-Test Langensteinbacher Version) part A and B [39] (see also S1 Table). Additional parts of the neuropsychological assessment are the CDT [35] and the LAT [34]. For a detailed description of the schedule see Table 1.

**Table 1:** Schedule of the neuropsychological assessment.

Test	Subtest	Duration in minutes
MMSE		10
Mini-SCL, PDQ		5
TQ Drive		10
LAT	S1	10
Test set CFD	S1	60
CDT		3
Total		98

Annotation. MMSE = Mini Mental Status Test; Mini-SCL = Mini-Symptom-Checklist; PDQ = Perceived Deficits Questionnaire; TQ Drive = Test Questionnaire Drive, unpublished questionnaire: Self assessment of driving experience and competence (see also S1 Questionnaire); LAT = Linien-Ausrichtungstest (German adaption of the Judgement of Line Orientation Test, JLO); CFD = Cognitive Functions Dementia; CDT = Clock-Drawing-Test.

#### 2.4.4 On-road assessment

The “Wasserburger Fahrverhaltensbeobachtung” (WAFAB), an on-road driving assessment, has been tried and tested in clinical practice over the last 10 years and method has been published (e.g. Brunnauer et al., 2015 [40]). It will be conducted by a certified driving instructor and a psychological technical assistant (PTA), who is experienced dealing with inpatients with neurological or psychiatric disorders. The driving instructor and the PTA will be blinded regarding the participant’s cognitive status and diagnoses. Participants will be requested to drive in a driving instruction vehicle on a predetermined route of approximately 50 kilometers length, which is segmented in narrowly defined time-proven observational spots. As a global measure every observation sequence will be rated on an 11-point Fitness-to-Drive-Scale (adapted from Neukum et al., 2003 [41]), which consists of three verbal categories and within each of them three to four numeric subcategories (normal 0–3, limited 4–6 and critical 7–10). In addition the driving errors will be rated by trained PTAs regarding total amount of errors, tactical errors with respect to longitudinal control (speed too high, inadequate speed, speed too low, time headway too low/tailgating), operational errors with respect to lateral control (lane departures/bad lane keeping, lateral distance to objects/vehicles too low) and cognitively based tactical errors (errors in changing/choosing lane, driving on impermissible lanes, securing behavior, communication, navigation errors) on determined observational spots; as an ancillary category critical situations (threats to other traffic participants, collisions) will be gathered. The driving instructor will fill out a 11-point Fitness-to-Drive-Scale comprising the categories speed behavior, distance keeping, lane usage, securing behavior, threatening behavior, communication, collisions, navigation errors as well as accident-prone situations and interference by the driving instructor. The whole on-road driving assessment will be recorded with the aid of a GS6000-A12 dashcam in order to have the possibility of post hoc evaluating ambiguous situations.

### 2.5 Outcome measures

#### 2.5.1 Primary endpoints

The primary outcomes comprise the results of the neuropsychological assessment in attention (intrinsic alertness, divided attention, processing speed), executive functions (working memory, cognitive flexibility) and visuospatial abilities (visuoconstruction, visual orientation). Results can be shown in percentile ranks and raw data as an aggregated score as well as a single score in every subtest. As mentioned before especially processing speed, cognitive flexibility and visual orientation ability are of utmost interest. The performance in the on-road assessment can be measured by means of an overall rating on an 11-point rating scale or on a more detailed level on the basis of tactical errors, operational errors or cognitively based tactical errors.

#### 2.5.2 Secondary endpoints

Secondary outcome variables are performances in neuropsychological domains such as expressive speech/language (lexical verbal fluency, semantic verbal fluency, object naming), and verbal long term memory (learning ability, short-term delayed recall, long-term delayed recall, recognition) also shown as an aggregated score (percentile rank) as well as a single score in every subtest. Other data–sociodemographic (age, gender, level of education, occupation etc.), clinical (record of diseases, medication etc.) and traffic-specific (years of traffic participation, crashes, fees etc.)–will be analyzed with respect to their influence on the prediction of driving behavior.

#### 2.5.3 Safety assessment, quality assurance and Ethics

The study will be conducted according to the Declaration of Helsinki and was approved by the Ethics Committee of the Medical Faculty of the Ludwig-Maximilians-University Munich. All participants have to give written informed consent prior to inclusion in the study. For privacy protection, participants will be identified using an identification code. Information such as participants’ names and addresses will be managed exclusively at the examination center and will not be provided to third parties. If it is necessary to provide experimental data to a joint research institution, this will be carefully protected using only the participants’ identification codes. The final results will be disseminated through peer-reviewed publications and various conferences.

At all times there will be contact to psychologically trained staff, which ensures fast communication of complaints and immediate response. Furthermore, the study procedure can be cancelled at any time. Risk of adverse events is considered low. By participating, participants will receive a free and professional assessment of their driving safety.

### 2.6 Sample size calculation

Studies examining the relation between driving ability and cognitive abilities show medium to large effect sizes for TMT (Part A and B) and tests on visual-spatial functions such as the Judgment of Line Orientation Test [14, 15, 20, 24]. Based on this we expect at least a medium effect size for the subtests TMT-L (Part A and B) and LAT in predicting on-road driving performance. Accordingly, the main analysis will be a multiple linear regression (fixed model, R^2^ deviation from zero) in which on-road driving performance is the outcome and the main test variables of TMT-L (Part A and B) and LAT are the three predictors. Given a power (1 - β) of 80%, an alpha-level of α = .05, and an effect size of f2 = 0.15, power analysis [42] indicated that the minimum sample size for detecting such an effect is N = 77.

### 2.7 Statistical analysis

The analyses will be performed by means of the computer software, IBM SPSS Statistics for Windows (version 25). We will calculate descriptive statistics for all predictor variables to obtain means and frequencies. The normality of sample distribution will be addressed using Shapiro-Wilk test. Equality of variance will be tested using Levene’s test, data sphericity using Mauchly’s test, and a Greenhouse–Geisser correction in case of the nonsphericity of the data. The effect sizes will be reported as the partial Eta square (η^2^) values and Cohen’s d. The alpha level is α = 0.05 and multiple comparisons will be corrected using Bonferroni. We will calculate multivariate analyses of variance with subsequent post-hoc-tests, construct multiple and logistic regression models, considering all neuropsychological variables as potential predictors, and conduct analyses of sensitivity and specificity.

## 3 Discussion

### 3.1 Clinical implications

In clinical practice counselling patients with respect to driving safety is of great relevance, not least because of legal constraints. Health status may have an influence on driving ability and studies suggest that the presence of particular cognitive impairments increases driving risks. Neurological and psychiatric diseases as well as medical conditions have a negative impact on driving status, especially if they are accompanied by distinct cognitive impairments [2]. Furthermore, driving cessation is often associated with decreased mobility, activity and independence. In a systematic review and meta-analysis Steel et al. (2014) [43] outlined that the lifetime prevalence of psychiatric illnesses add up to 29.2%. These diseases are accompanied with impairment of different functions relevant for driving and therefore lead to a 2.1 up to 5.0 times higher risk of accidents in road traffic [3]. Cognitive impairment or rather impairment of driving ability derives not only from the disease itself, but also from drug treatment. Therefore, road safety under pharmacological treatment is of great relevance for patients with a psychiatric disease.

However, there is still a gap between scientific results and clinical practice, which is hindered by the lack of validated cut-off scores for most research findings. Molnar et al. (2006) [13] stated, that it is impossible to employ tests in a standardized fashion in the forefront of clinical practice without validated cut-off-scores. Only a few studies yielded results that could distinguish between fit and unfit to drive. Recommendations implicate a classification accuracy of about 80–90% to ensure a consistent precise prediction [44]. In contrast to the previous dichotomization between safe and unsafe drivers, a trichotomization is suggested, that would divide drivers into “safe”, “uncertain” and “unsafe” drivers [13]. The intermediate category “uncertain” would advise practitioners and patients to undertake a more precise assessment, for example by means of an on-road driving test, for the purpose of precise categorization. In developing such a screening-tool, highest priority should be given to its efficient implementation in clinical routines. With that said it must not be resource-intensive regarding to time and personnel, which suggests a tablet- or computer-based application.

This project, which aims to demonstrate high predictive validity of a screening tool concerning driving safety of cognitively impaired older people, is highly practice-oriented due to the following reasons: On the one hand the number of seniors participating in road traffic will continuously increase in the following years as a result of the demographic change. On the other hand there is great uncertainty on behalf of medical practitioners especially in the ambulant setting regarding adequate mobility consulting. It is well known that driving cessation is often associated with decreased mobility, activity and independence. On the basis of evidence-based predictive neuropsychological domains (attention, executive functions, visuospatial capacities), it is actually possible to fulfill statutory provisions such as obligation to inform on the part of the practitioner and responsibility/duty to precaution on the part of the patient. Besides, being obliged to inform their patients appropriately concerning implications of illness and medical treatment on motor traffic, general physicians and psychologists are then put in the position to consider compensation opportunities that enable restricted driving ability [9]. With the aid of this screening tool, which can be applied tablet-based, low-treshold and therefore time-saving without being personnel intensive, we can counteract this problem. A calculated so-called “fit-value” which entails ideal ranges and variable weights, leads to a trichotomous output in “safe to drive”, “needs further assessment” and “unsafe to drive” and hence guarantees a very user-friendly interpretation of the results. Thus, having valid, reliable, time economical and simply interpretable screening-tools on hand to counsel patients is of great relevance. With this study we expect to evaluate a screening tool to help clinicians to counsel patients with respect to driving ability.

### 3.2 Limitations

Possible limitations of our study are: (i) Implementing an on-road assessment, one typical limitation is the reliability i.e. the inability to control variables such as traffic flow, road conditions, and other drivers’ behavior. This may increase errors in the on-road test results and therefore decrease the strength of their relationship with neuropsychological tests. (ii) The study process may be very tiring especially for the experimental group of cognitively impaired participants owing to a study duration of three and a half hours. The appropriateness could be questioned. (iii) There may be a selection bias as only inpatients who consider themselves to be able to participate in a long and arduous procedure will be included. (iv) Inpatients will be treated with medication and psychological therapies as well as supportive therapies (occupational and sport therapy) in accord with clinical considerations, which cannot be controlled. (v) By using MMSE as one criteria for group allocation, only patients with rather severe cognitive deficits will be included due to the lack of MMSE’s sensitivity to mild cognitive impairment. Nonetheless we will use this screening by virtue of its high prevalence in clinical practice.

## Supporting information

S1 Checklist SPIRIT 2013 checklist: Recommended items to address in a clinical trial protocol and related documents*. (DOCX)

S1 Table Dimensions and subtests of the test-set CFD. (PDF)

S1 Questionnaire TQ-Drive. (PDF)

S1 File (DOCX)

S2 File (DOCX)
