# Supplementary material for: Protocol for the conceptualization and evaluation of a screening-tool for fitness-to-drive assessment in older people with cognitive impairment
Source: PLoS One. 2021 Sep 1;16(9):e0256262. doi: 10.1371/journal.pone.0256262 (PMC8409688; doi:10.1371/journal.pone.0256262)
Supplement: S1 Checklist — (DOCX) [file pone.0256262.s001.docx]

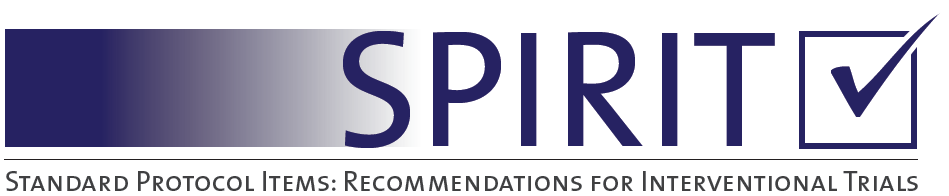


SPIRIT 2013 Checklist: Recommended items to address in a clinical trial protocol and related documents*

| **Section/item** | **ItemNo** | **Description** |
| --- | --- | --- |
| **Administrative information** | | |
| Title | 1 | Protocol for the conceptualization and evaluation of a screening-tool for fitness-to-drive assessment in elders with cognitive impairment |
| Trial registration | 2a | German Clinical Trials Register |
|  | 2b | All items from the World Health Organization Trial Registration Data Set |
| Protocol version | 3 | **Issue Date:** 16 Nov 2020 **Author(s):** L.Z. |
| Funding | 4 | The study was founded by an unrestriced grant from Schuhfried GmbH. None of the authors received an award. |
| Roles and responsibilities | 5a | Leonhard Zellner^1¶^*, Florian Herpich^1¶^, David Brieber^2&^, Margit Herle^2&^, Peter Zwanzger^1,3&^, Alexander Brunnauer^1,3¶^  ^1^kbo-Inn-Salzach-Klinikum, Psychiatric Hospital, Wasserburg am Inn, Germany  ^2^Schuhfried GmbH, Mödling, Austria  ^3^Department of Psychiatry and Psychotherapy, Ludwig-Maximilians University Munich, Germany  * Corresponding author.   - Leonhard Zellner (LZ), ORCID-iD: 0000-0002-3129-8087, **Conceptualization, Investigation, Methodology, Writing – Original Draft Preparation**   ^¶^These authors contributed equally to this work:   - Leonhard Zellner (LZ) - Florian Herpich (FH): Formal Analysis, **Writing – Review & Editing** - Alexander Brunnauer (AB): **Project Administration, Conceptualization, Methodology, Funding Acquisition, Resources, Writing – Review & Editing**   ^&^These authors also contributed equally to this work:   - David Brieber (DB): Formal Analysis, Data Curation, Software, **Writing – Review & Editing** - Margit Herle (MH): Formal Analysis, Data Curation, Software, **Writing – Review & Editing** - Peter Zwanzger (PZ): **Project Administration, Writing – Review & Editing** |
|  | 5b | Schuhfried GmbH, Hyrtlstrasse 45, 2340 Moedling, Austria |
|  | 5c | This funding source had no role in the design of this study and will not have any role during its execution, analyses, interpretation of the data, or decision to submit results |
|  | 5d | Not applicable |
| **Introduction** |  |  |
| Background and rationale | 6a | Introduction: Due to aging and health status people may be subjected to a decrease of cognitive ability and subsequently also a decline of driving safety. On the other hand there is a lack of valid and economically applicable instruments to assess driving performance. Objective: The study is designed to develop a valid screening-tool for fitness-to-drive assessment in elders with cognitive impairment externally validated on the basis of on-road driving performance.  Methods: In a monocentric, non-randomized cross-sectional trial cognitive functioning and onroad-driving-behavior of elderly drivers will be assessed. Forty participants with cognitive impairment of different etiology and 40 healthy controls will undergo an extensive neuropsychological assessment. Additionally, an on-road driving assessment for external validation of fitness to drive will be carried out. Primary outcome measures will be performance in attention, executive functions and visuospatial tasks that will be validated with respect to performance on the on-road-driving-test. Secondary outcome measures will be sociodemographic, clinical- and driving characteristics to systematically examine their influence on the prediction of driving behavior.  Discussion: In clinical practice counselling patients with respect to driving safety is of great relevance. Thus, having valid, reliable, time economical and easily interpretable screening-tools on hand to counsel patients is of great relevance for practitioners. |
|  | 6b | Not applicable |
| Objectives | 7 | Hypotheses: We assume that cognitively impaired and healthy subjects will differ significantly regarding on-road driving performance. Moreover among assessed cognitive domains we expect especially visuospatial performance, attention and executive functions to predict driving ability and the newly developed screening-tool to differentiate with high diagnostical accuracy between safe and unsafe drivers. Besides it is expected that driving competence will be additionally predicted by factors like age, driving experience in years and mileage per year.  Objective: The study is designed to develop a valid screening-tool for fitness-to-drive assessment in elders with cognitive impairment externally validated on the basis of on-road driving performance. |
| Trial design | 8 | Non-interventional, observational study; Allocation: non-randomized controlled trial. |
| **Methods: Participants, interventions, and outcomes** | | |
| Study setting | 9 | Academic hospital (monocentric); data will only be collected in this institution (Germany). |
| Eligibility criteria | 10 | Inclusion criteria: Age ≥ 50 years; valid driver license; constant participation in road traffic; good German language skills; MMSE ≥18 and <27 for the cognitively impaired patients: 20 patients with organic, including symptomatic disorders (F00-F09), 10 with affective disorders (F30-F39), 10 with schizophrenia, schizotypal or delusional disorders (F20-F29) or neurotic, stress-related and somatoform disorders (F40-F48); MMSE ≥ 27 for the healthy controls  Exclusion criteria: Moderate to severe dementia (MMSE < 18); severe psychiatric, neurological or internistic disease; no current radio- or chemotherapy; visual impairment (visual acuity worse than 60%; visual field <140˚, double vision, hemi-neglect) |
| Interventions | 11a | Assessment of psychopathology and extensive neuropsychological examination of cognitively impaired elders over 50 years (experimental group) by means of standardized test procedures. In order to assess sociodemographic, clinical as well as driving related data we will use standardized questionnaires and interviews and rating scales (Mini-Mental-State-Examination, MMSE; Mini-Symptom-Checklist, Mini-SCL; Perceived Deficits Questionnaire, PDQ). The neuropsychological assessment comprises the test battery Cognitive Functions Dementia (CFD), the Line Orientation Test (LAT, abbreviation based on the German test name) and the Clock-Drawing-Test (CDT). The following on-road assessment will be conducted by a certified driving instructor and a psychological technical assistant (PTA). The driving instructor and the PTA will be blinded regarding the participant’s cognitive status and diagnoses. Participants will be requested to drive in a driving instruction vehicle on a predetermined route of approximately 50 kilometers length and will be rated on an 11-point Fitness-to-Drive-Scale.  Control group (healthy persons) analogous to experimental group. |
|  | 11b | Not applicable |
|  | 11c | Not applicable |
|  | 11d | Not applicable |
| Outcomes | 12 | The primary outcomes comprise the results of the neuropsychological assessment in attention (intrinsic alertness, divided attention, processing speed), visuospatial abilities (visuoconstruction, visual orientation) and executive functions (working memory, cognitive flexibility). The test-set Cognitive Functions Dementia (CFD), the Judgement-of-Line-Orientation-Test (JLO) and Clock-Drawing-Test (CDT) are applied. Furthermore the performance in the on-road assessment can be measured by means of an overall rating on an 11-point rating scale or on a more detailed level on the basis of tactical errors, operational errors or cognitively based tactical errors.  Secondary outcome variables are performances in neuropsychological domains such as expressive speech/language (lexical verbal fluency, semantic verbal fluency, object naming), and verbal long term memory (learning ability, short-term delayed recall, long-term delayed recall, recognition) acquired by the test-set Cognitive Functions Dementia (CFD). Anamnestic data – sociodemographic (age, gender, level of education, occupation etc.), clinical (record of diseases, medication etc.) and traffic-specific (years of traffic participation, crashes, fees etc.) – will be analyzed with respect to their influence on the prediction of driving behavior. Standardized questionnaires, interviews and Rating scales (Mini-Mental-State-Examination, MMSE; Mini-Symptom-Checklist, Mini-SCL; Perceived Deficits Questionnaire, PDQ) will be used. |
| Participant timeline | 13 |  |
| Sample size | 14 | The calculated sample size of N = 80 results as follows: Supposing a power (1 – β) of 80% and an alpha-level of α = .05 a sample size of N = 77 is necessary to detect an average effect calculating a multiple regression with three predictors (TMT-L part A, part B and LAT). In case of early dropouts we calculate a sample size of N=80 for recruitment. |
| Recruitment | 15 | Recruitment takes place by means of mail to the staff of the kbo-Inn-Salzach-Clinic in Wasserburg/Inn, displays on the clinic campus as well as advertisement in relevant magazines and information-flyers. It is being requested to make interested friends and other contacts aware of the study. Besides we inform patients of different diagnosis groups F00. F20 and F30 via individual departments of the Inn-Salzach-Clinic. |
| **Methods: Assignment of interventions (for controlled trials)** | | |
| Allocation: |  |  |
| Sequence generation | 16a | Group allocation is conducted via MMSE-score:   - Healthy subjects (Control group): MMSE ≥27 - Cognitively impaired subjects (Experimental group) : MMSE ≥18 and <27 |
| Allocation concealment mechanism | 16b | Not applicable |
| Implementation | 16c | Not applicable |
| Blinding (masking) | 17a | Open (masking not used) |
|  | 17b | Not applicable |
| **Methods: Data collection, management, and analysis** | | |
| Data collection methods | 18a | Assessment of psychopathology and extensive neuropsychological examination of cognitively impaired elders over 50 years (experimental group) by means of standardized test procedures. In order to assess sociodemographic, clinical as well as driving related data we will use standardized questionnaires and interviews and rating scales (Mini-Mental-State-Examination, MMSE; Mini-Symptom-Checklist, Mini-SCL; Perceived Deficits Questionnaire, PDQ). The neuropsychological assessment comprises the test battery Cognitive Functions Dementia (CFD), the Line Orientation Test (LAT, abbreviation based on the German test name) and the Clock-Drawing-Test (CDT). The following on-road assessment will be conducted by a certified driving instructor and a psychological technical assistant (PTA). The driving instructor and the PTA will be blinded regarding the participant’s cognitive status and diagnoses. Participants will be requested to drive in a driving instruction vehicle on a predetermined route of approximately 50 kilometers length and will be rated on an 11-point Fitness-to-Drive-Scale. |
|  | 18b | Not applicable |
| Data management | 19 | All data gathered in the context of the study exclusively serve research purposes, are kept in confidence (stored in a locker) and are anonymised irreversibly after elicitation plus saved for an indefinite period. The results will be analysed scientifically after finalization of the study and will be published in anonymised manner which prevents inference to the participant’s person. See also Item 27. |
| Statistical methods | 20a | The analyses will be performed by means of the computer software, IBM SPSS Statistics for Windows (version 25). We will calculate descriptive statistics for all predictor variables to obtain means and frequencies. The normality of sample distribution will be addressed using Shapiro-Wilk test. Equality of variance will be tested using Levene's test, data sphericity using Mauchly’s test, and a Greenhouse–Geisser correction in case of the nonsphericity of the data. The effect sizes will be reported as the partial Eta square (η^2^) values and Cohen's d. The alpha level is α = 0.05 and multiple comparisons will be corrected using Bonferroni. We will calculate multivariate analyses of variance with subsequent post-hoc-tests, construct multiple (logistic) regression models, considering all neuropsychological variables as potential predictors, and conduct analyses of sensitivity and specificity. |
|  | 20b | Not applicable |
|  | 20c | Not applicable |
| **Methods: Monitoring** | | |
| Data monitoring | 21a | DMC is not needed because of known minimal risks (see also Item 22). |
|  | 21b | Not applicable |
| Harms | 22 | At all times there will be contact to psychologically trained staff, which ensures fast communication of complaints and immediate response. Furthermore, the study procedure can be cancelled at any time. The occurrence probability of further risks is estimated low. In context of their participation the participants receive a free and professional assessment of their driving safety. |
| Auditing | 23 | Not applicable |
| **Ethics and dissemination** | | |
| Research ethics approval | 24 | Ethics approval was obtained from the Ethics Committee at the Ludwig-Maximilians-University Munich (project number: 18-640). Moreover the study is registered at the German Register for Clinical Studies, DRKS (registration code: DRKS00023549) |
| Protocol amendments | 25 | Not applicable |
| Consent or assent | 26a | The principal investigator will obtain informed consent in written form from all study participants. |
|  | 26b | Not applicable |
| Confidentiality | 27 | For privacy protection, participants will be identified using an identification code. Information such as participants’ names and addresses will be managed exclusively at the examination center and will not be provided to third parties. Experimental data necessary to provide to a joint research institution will be carefully protected using only the participant’s identification codes. The final results will be disseminated through peer-reviewed publications and various conferences (see also Item 19). |
| Declaration of interests | 28 | There are no financial and other competing interests for principal investigators for the overall trial and each study site. |
| Access to data | 29 | Statement of who will have access to the final trial dataset, and disclosure of contractual agreements that limit such access for investigators |
| Ancillary and post-trial care | 30 | Not applicable |
| Dissemination policy | 31a | The final results will be disseminated through peer-reviewed publications and various conferences. |
|  | 31b | See Item 5a. |
|  | 31c | Not applicable |
| **Appendices** |  |  |
| Informed consent materials | 32 | Model consent form and other related documentation given to participants and authorised surrogates |
| Biological specimens | 33 | Not applicable |

*It is strongly recommended that this checklist be read in conjunction with the SPIRIT 2013 Explanation & Elaboration for important clarification on the items. Amendments to the protocol should be tracked and dated. The SPIRIT checklist is copyrighted by the SPIRIT Group under the Creative Commons “[Attribution-NonCommercial-NoDerivs 3.0 Unported](http://www.creativecommons.org/licenses/by-nc-nd/3.0/)” license.
