# Supplementary material for: Protocol for the conceptualization and evaluation of a screening-tool for fitness-to-drive assessment in older people with cognitive impairment
Source: PLoS One. 2021 Sep 1;16(9):e0256262. doi: 10.1371/journal.pone.0256262 (PMC8409688; doi:10.1371/journal.pone.0256262)
Supplement: S1 Table — (PDF) [file pone.0256262.s002.pdf]

**Dimension**	**Test**	**Subtest**	**Duration in minutes**
Semantic fluency	WIWO	S1	3
Lexical fluency	WIWO	S3	4
Learning ability	AWLT (Subtest 1)	S1	7
Alertness (intrinsic-visual)	WAFA	S2	2
Brief delayed recall	AWLT (Subtest 2)	S1	2
Divided attention	WAFG	S3	9
Processing speed	TMT	S1	1
Cognitive flexibility	TMT	S1	1
Working memory, spatial	CORSI	S7	8
Long delayed recall and discrimination	AWLT (Subtests 3 und 4)	S1	4
Object naming	WOBT	S1	5
Visuo-construction	VISCO	S3	7
