# Supplementary material for: Protocol for the conceptualization and evaluation of a screening-tool for fitness-to-drive assessment in older people with cognitive impairment
Source: PLoS One. 2021 Sep 1;16(9):e0256262. doi: 10.1371/journal.pone.0256262 (PMC8409688; doi:10.1371/journal.pone.0256262)
Supplement: S1 Questionnaire — (PDF) [file pone.0256262.s003.pdf]

Below you will be asked a few questions that relate to your driving behavior. The information you provide will be used exclusively for further analyses as part of the scientific study and will, of course, be treated confidentially and anonymously. Please answer all questions honestly and as accurately as possible.

1. How many years have you held your Class B driver's license?

   ____

2. What car do you currently drive?

   - ☐ Automatik
   - ☐ Manual transmission
   - ☐ Both

3. How many kilometers do you drive on average per month?
   It is sufficient if you provide a rough estimate here.

   ____

4. How often do you drive by car?

   - ☐ Less than 1x per week
   - ☐ 1-2x per week
   - ☐ 3-4x per week
   - ☐ 5-6x per week
   - ☐ Daily

5. How often do you drive by car **in a small town (e.g. Wasserburg am Inn)?**

   - ☐ Less than 1x per week
   - ☐ 1-2x per week
   - ☐ 3-4x per week
   - ☐ 5-6x per week
   - ☐ Daily

6. How often do you drive by car **in a big city (e.g. Munich)?**

   - ☐ Less than 1x per week
   - ☐ 1-2x per week
   - ☐ 3-4x per week
   - ☐ 5-6x per week
   - ☐ Daily

7. How often do you drive by car **on a highway?**

   - ☐ Less than 1x per week
   - ☐ 1-2x per week
   - ☐ 3-4x per week

- ☐ 5-6x per week
- ☐ Daily

8. How often do you drive by car **on a country road?**

- ☐ Less than 1x per week
- ☐ 1-2x per week
- ☐ 3-4x per week
- ☐ 5-6x per week
- ☐ Daily

9. How often do you drive **to run errands or meet obligations? (e.g. physician, shopping, bank)?**

- ☐ Less than 1x per week
- ☐ 1-2x per week
- ☐ 3-4x per week
- ☐ 5-6x per week
- ☐ Daily

10. How often do you drive **to work?**

- ☐ Less than 1x per week
- ☐ 1-2x per week
- ☐ 3-4x per week
- ☐ 5-6x per week
- ☐ Daily

11. How often do you drive **to visit friends or family?**

- ☐ Less than 1x per week
- ☐ 1-2x per week
- ☐ 3-4x per week
- ☐ 5-6x per week
- ☐ Daily

12. How often do you drive **to sports or other volunteer activities (e.g., volunteer work)?**

- ☐ Less than 1x per week
- ☐ 1-2x per week
- ☐ 3-4x per week
- ☐ 5-6x per week
- ☐ Daily

13. How often do you drive for **day trips or vacations?**

- ☐ Less than 1x per week
- ☐ 1-2x per week
- ☐ 3-4x per week
- ☐ 5-6x per week
- ☐ Daily

14. How often do you drive by car **for the pleasure of driving?**

- ☐ Less than 1x per week
- ☐ 1-2x per week
- ☐ 3-4x per week
- ☐ 5-6x per week
- ☐ Daily

16. Do you have difficulties **driving in heavy traffic?**

- ☐ Very rare / Never
- ☐ Rather rare
- ☐ Sometimes
- ☐ Rather frequent
- ☐ Very frequent

15. Do you have difficulty **driving in poor weather or visibility conditions? (e.g. rain)**

- ☐ Very rare / Never
- ☐ Rather rare
- ☐ Sometimes
- ☐ Rather frequent
- ☐ Very frequent

17. Do you have difficulties **with longer trips (over 50 kilometers)?**

- ☐ Very rare / Never
- ☐ Rather rare
- ☐ Sometimes
- ☐ Rather frequent
- ☐ Very frequent

19. Do you have difficulties with **driving in the dark or at dusk?**

- ☐ Very rare / Never
- ☐ Rather rare
- ☐ Sometimes
- ☐ Rather frequent
- ☐ Very frequent

18. Do you have difficulties **driving on unfamiliar routes?**

- ☐ Very rare / Never
- ☐ Rather rare
- ☐ Sometimes
- ☐ Rather frequent
- ☐ Very frequent

20. Do you have difficulties **driving on highways?**

- ☐ Very rare / Never
- ☐ Rather rare
- ☐ Sometimes
- ☐ Rather frequent
- ☐ Very frequent

21. Do you have difficulties **driving on country roads?**

- ☐ Very rare / Never
- ☐ Rather rare
- ☐ Sometimes
- ☐ Rather frequent
- ☐ Very frequent

22. Do you have difficulties **driving in city traffic?**

- ☐ Very rare / Never
- ☐ Rather rare
- ☐ Sometimes
- ☐ Rather frequent
- ☐ Very frequent

22. Do you have difficulties **driving with more than one passenger? (e.g. your grandchildren)**

- ☐ Very rare / Never
- ☐ Rather rare
- ☐ Sometimes
- ☐ Rather frequent
- ☐ Very frequent

23. Do you avoid **driving in poor weather or visibility conditions? (e.g. rain)**

- ☐ Very rare / Never
- ☐ Rather rare
- ☐ Sometimes
- ☐ Rather frequent
- ☐ Very frequent

24. Do you avoid **driving when traffic is heavy?**

- ☐ Very rare / Never
- ☐ Rather rare
- ☐ Sometimes
- ☐ Rather frequent
- ☐ Very frequent

25. Do you avoid **longer trips (over 50 kilometers)?**

- ☐ Very rare / Never

- ☐ Rather rare
- ☐ Sometimes
- ☐ Rather frequent
- ☐ Very frequent

26. Do you avoid **driving on unfamiliar routes?**

- ☐ Very rare / Never
- ☐ Rather rare
- ☐ Sometimes
- ☐ Rather frequent
- ☐ Very frequent

27. Do you avoid **driving in the dark and at dusk?**

- ☐ Very rare / Never
- ☐ Rather rare
- ☐ Sometimes
- ☐ Rather frequent
- ☐ Very frequent

28. Do you avoid **driving on highways?**

- ☐ Very rare / Never
- ☐ Rather rare
- ☐ Sometimes
- ☐ Rather frequent
- ☐ Very frequent

29. Do you avoid **driving on country roads?**

- ☐ Very rare / Never
- ☐ Rather rare
- ☐ Sometimes
- ☐ Rather frequent
- ☐ Very frequent

30. Do you avoid **driving in city traffic?**

- ☐ Very rare / Never
- ☐ Rather rare
- ☐ Sometimes
- ☐ Rather frequent
- ☐ Very frequent

31. Do you avoid **driving with more than one passenger? (e.g. your grandchildren)?**

- ☐ Very rare / Never
- ☐ Rather rare
- ☐ Sometimes

- ☐ Rather frequent
- ☐ Very frequent

32. How do you rate your **driving ability** compared to other drivers your age?

- ☐ Below average
- ☐ Rather below average
- ☐ Average
- ☐ Rather above average
- ☐ Above average

33. How would you rate your **ability to maintain your concentration for an extended period of time** compared to other drivers?

- ☐ Below average
- ☐ Rather below average
- ☐ Average
- ☐ Rather above average
- ☐ Above average

34. How would you rate your **ability to consciously pay attention to multiple traffic aspects at once** compared to other drivers?

- ☐ Below average
- ☐ Rather below average
- ☐ Average
- ☐ Rather above average
- ☐ Above average

35. How do you rate your **ability** to react compared to other drivers **(e.g. in dangerous situations)?**

- ☐ Below average
- ☐ Rather below average
- ☐ Average
- ☐ Rather above average
- ☐ Above average

36. How good do you consider your **ability to keep an overview in dangerous traffic situations** compared to other drivers?

- ☐ Below average
- ☐ Rather below average
- ☐ Average
- ☐ Rather above average
- ☐ Above average

37. How good do you consider your **ability to focus on traffic events even when distracted (e.g., by other passengers or the radio)** compared to other drivers?

- ☐ Below average

- ☐ Rather below average
- ☐ Average
- ☐ Rather above average
- ☐ Above average

38. How do you rate yourself in terms of your **safety** as a driver?

- ☐ Unsafe driver
- ☐ Rather unsafe driver
- ☐ Average safe driver
- ☐ Rather safe driver
- ☐ Safe driver

Im Folgenden werden Ihnen einige Fragen gestellt, die sich auf Ihr Fahrverhalten beziehen. Ihre Angaben dienen ausschließlich für weitergehende Analysen im Rahmen der wissenschaftlichen Untersuchung und werden selbstverständlich vertraulich und anonym behandelt. Bitte beantworten Sie alle Fragen ehrlich und so genau wie möglich.

1. Seit wie vielen Jahren besitzen Sie Ihren Führerschein der Klasse B?

   ____

2. Welches Auto fahren Sie derzeit?

   - ☐ Automatik
   - ☐ Schaltgetriebe
   - ☐ Beides

3. Wie viele Kilometer fahren Sie durchschnittlich im Monat?
   Es reicht aus, wenn Sie hier eine grobe Schätzung angeben.

   ____

4. Wie häufig fahren Sie mit dem Auto?

   - ☐ Weniger als 1x pro Woche
   - ☐ 1-2x pro Woche
   - ☐ 3-4x pro Woche
   - ☐ 5-6x pro Woche
   - ☐ Täglich

5. Wie häufig fahren Sie mit dem Auto **in einer Kleinstadt (z.B. Wasserburg am Inn)?**

   - ☐ Weniger als 1x pro Woche
   - ☐ 1-2x pro Woche
   - ☐ 3-4x pro Woche
   - ☐ 5-6x pro Woche
   - ☐ Täglich

6. Wie häufig fahren Sie mit dem Auto **in einer Großstadt (z.B. München)?**

   - ☐ Weniger als 1x pro Woche
   - ☐ 1-2x pro Woche
   - ☐ 3-4x pro Woche
   - ☐ 5-6x pro Woche
   - ☐ Täglich

7. Wie häufig fahren Sie mit dem Auto **auf einer Autobahn?**

   - ☐ Weniger als 1x pro Woche
   - ☐ 1-2x pro Woche
   - ☐ 3-4x pro Woche

- ☐ 5-6x pro Woche
- ☐ Täglich

8. Wie häufig fahren Sie mit dem Auto **auf einer Landstraße?**

- ☐ Weniger als 1x pro Woche
- ☐ 1-2x pro Woche
- ☐ 3-4x pro Woche
- ☐ 5-6x pro Woche
- ☐ Täglich

9. Wie häufig fahren Sie mit dem Auto **um Besorgungen zu erledigen oder Verpflichtungen nachzugehen? (z.B. Arzt, Einkauf, Bank)?**

- ☐ Weniger als 1x pro Woche
- ☐ 1-2x pro Woche
- ☐ 3-4x pro Woche
- ☐ 5-6x pro Woche
- ☐ Täglich

10. Wie häufig fahren Sie mit dem Auto **zur Arbeit?**

- ☐ Weniger als 1x pro Woche
- ☐ 1-2x pro Woche
- ☐ 3-4x pro Woche
- ☐ 5-6x pro Woche
- ☐ Täglich

11. Wie häufig fahren Sie mit dem Auto **um Freunde oder Familie zu besuchen?**

- ☐ Weniger als 1x pro Woche
- ☐ 1-2x pro Woche
- ☐ 3-4x pro Woche
- ☐ 5-6x pro Woche
- ☐ Täglich

12. Wie häufig fahren Sie mit dem Auto **zum Sport oder zu sonstigen freiwilligen Aktivitäten (z.B. ehrenamtliche Tätigkeiten)?**

- ☐ Weniger als 1x pro Woche
- ☐ 1-2x pro Woche
- ☐ 3-4x pro Woche
- ☐ 5-6x pro Woche
- ☐ Täglich

13. Wie häufig fahren Sie mit dem Auto **zu Tagesausflügen oder in den Urlaub?**

- ☐ Weniger als 1x pro Woche
- ☐ 1-2x pro Woche
- ☐ 3-4x pro Woche

- ☐ 5-6x pro Woche
- ☐ Täglich

14. Wie häufig fahren Sie mit dem Auto **aus Lust am Autofahren?**

- ☐ Weniger als 1x pro Woche
- ☐ 1-2x pro Woche
- ☐ 3-4x pro Woche
- ☐ 5-6x pro Woche
- ☐ Täglich

16. Haben Sie Schwierigkeiten mit **Fahrten bei hohem Verkehrsaufkommen?**

- ☐ Sehr selten / Nie
- ☐ Eher selten
- ☐ Manchmal
- ☐ Eher häufig
- ☐ Sehr häufig

15. Haben Sie Schwierigkeiten mit **Fahrten bei schlechten Witterungs- oder Sichtverhältnissen? (z.B. Regen)**

- ☐ Sehr selten / Nie
- ☐ Eher selten
- ☐ Manchmal
- ☐ Eher häufig
- ☐ Sehr häufig

17. Haben Sie Schwierigkeiten mit **längeren Fahrten (über 50 Kilometer)?**

- ☐ Sehr selten / Nie
- ☐ Eher selten
- ☐ Manchmal
- ☐ Eher häufig
- ☐ Sehr häufig

19. Haben Sie Schwierigkeiten mit **Fahrten bei Dunkelheit und Dämmerung?**

- ☐ Sehr selten / Nie
- ☐ Eher selten
- ☐ Manchmal
- ☐ Eher häufig
- ☐ Sehr häufig

18. Haben Sie Schwierigkeiten mit **Fahrten auf unbekannten Strecken?**

- ☐ Sehr selten / Nie
- ☐ Eher selten
- ☐ Manchmal

- ☐ Eher häufig
- ☐ Sehr häufig

20. Haben Sie Schwierigkeiten mit **Fahrten auf Autobahnen?**

- ☐ Sehr selten / Nie
- ☐ Eher selten
- ☐ Manchmal
- ☐ Eher häufig
- ☐ Sehr häufig

21. Haben Sie Schwierigkeiten mit **Fahrten auf Landstraßen?**

- ☐ Sehr selten / Nie
- ☐ Eher selten
- ☐ Manchmal
- ☐ Eher häufig
- ☐ Sehr häufig

22. Haben Sie Schwierigkeiten mit **Fahrten im Stadtverkehr?**

- ☐ Sehr selten / Nie
- ☐ Eher selten
- ☐ Manchmal
- ☐ Eher häufig
- ☐ Sehr häufig

22. Haben Sie Schwierigkeiten mit **Fahrten mit mehr als einem Mitfahrer (z.B. mit Ihren Enkelkindern)?**

- ☐ Sehr selten / Nie
- ☐ Eher selten
- ☐ Manchmal
- ☐ Eher häufig
- ☐ Sehr häufig

23. Vermeiden Sie **Fahrten bei schlechten Witterungs- oder Sichtverhältnissen? (z.B. Regen)**

- ☐ Sehr selten / Nie
- ☐ Eher selten
- ☐ Manchmal
- ☐ Eher häufig
- ☐ Sehr häufig

24. Vermeiden Sie **Fahrten bei hohem Verkehrsaufkommen?**

- ☐ Sehr selten / Nie
- ☐ Eher selten
- ☐ Manchmal

- ☐ Eher häufig
- ☐ Sehr häufig

25. Vermeiden Sie **längere Fahrten (über 50 Kilometer)?**

- ☐ Sehr selten / Nie
- ☐ Eher selten
- ☐ Manchmal
- ☐ Eher häufig
- ☐ Sehr häufig

26. Vermeiden Sie **Fahrten auf unbekannten Strecken?**

- ☐ Sehr selten / Nie
- ☐ Eher selten
- ☐ Manchmal
- ☐ Eher häufig
- ☐ Sehr häufig

27. Vermeiden Sie **Fahrten bei Dunkelheit und Dämmerung?**

- ☐ Sehr selten / Nie
- ☐ Eher selten
- ☐ Manchmal
- ☐ Eher häufig
- ☐ Sehr häufig

28. Vermeiden Sie **Fahrten auf Autobahnen?**

- ☐ Sehr selten / Nie
- ☐ Eher selten
- ☐ Manchmal
- ☐ Eher häufig
- ☐ Sehr häufig

29. Vermeiden Sie **Fahrten auf Landstraßen?**

- ☐ Sehr selten / Nie
- ☐ Eher selten
- ☐ Manchmal
- ☐ Eher häufig
- ☐ Sehr häufig

30. Vermeiden Sie **Fahrten im Stadtverkehr?**

- ☐ Sehr selten / Nie
- ☐ Eher selten
- ☐ Manchmal

- ☐ Eher häufig
- ☐ Sehr häufig

31. Vermeiden Sie **Fahrten mit mehr als einem Mitfahrer (z.B. mit Ihren Enkelkindern)?**

- ☐ Sehr selten / Nie
- ☐ Eher selten
- ☐ Manchmal
- ☐ Eher häufig
- ☐ Sehr häufig

32. Wie schätzen Sie ihr **Fahrkönnen** im Vergleich zu anderen Autofahrern Ihres Alters ein?

- ☐ Unterdurchschnittlich
- ☐ Eher unterdurchschnittlich
- ☐ Durchschnittlich
- ☐ Eher überdurchschnittlich
- ☐ Überdurchschnittlich

33. Wie schätzen Sie Ihre **Fähigkeit, Ihre Konzentration über einen längeren Zeitraum aufrecht zu erhalten**, im Vergleich zu anderen Autofahrern ein?

- ☐ Unterdurchschnittlich
- ☐ Eher unterdurchschnittlich
- ☐ Durchschnittlich
- ☐ Eher überdurchschnittlich
- ☐ Überdurchschnittlich

34. Wie schätzen Sie Ihre **Fähigkeit, bewusst auf mehrere Verkehrsaspekte gleichzeitig zu achten**, im Vergleich zu anderen Autofahrern ein?

- ☐ Unterdurchschnittlich
- ☐ Eher unterdurchschnittlich
- ☐ Durchschnittlich
- ☐ Eher überdurchschnittlich
- ☐ Überdurchschnittlich

35. Wie schätzen Sie Ihre **Reaktionsfähigkeit (z.B. in Gefahrensituationen)** im Vergleich zu anderen Autofahrern ein?

- ☐ Unterdurchschnittlich
- ☐ Eher unterdurchschnittlich
- ☐ Durchschnittlich
- ☐ Eher überdurchschnittlich
- ☐ Überdurchschnittlich

36. Wie schätzen Sie Ihre **Fähigkeit, in Verkehrssituationen den Überblick zu behalten**, im Vergleich zu anderen Autofahrern ein?

- ☐ Unterdurchschnittlich
- ☐ Eher unterdurchschnittlich
- ☐ Durchschnittlich
- ☐ Eher überdurchschnittlich
- ☐ Überdurchschnittlich

37. Wie schätzen Sie Ihre **Fähigkeit, auch bei Ablenkung (z.B. durch andere Mitfahrer oder das Radio) auf das Verkehrsgeschehen zu fokussieren**, im Vergleich zu anderen Autofahrern ein?

- ☐ Unterdurchschnittlich
- ☐ Eher unterdurchschnittlich
- ☐ Durchschnittlich
- ☐ Eher überdurchschnittlich
- ☐ Überdurchschnittlich

38. Wie schätzen Sie sich hinsichtlich Ihrer **Sicherheit** als Autofahrer ein?

- ☐ Unsicherer Fahrer
- ☐ Eher unsicherer Fahrer
- ☐ Durchschnittlich sicherer Fahrer
- ☐ Eher sicherer Fahrer
- ☐ Sicherer Fahrer
