# Supplementary material for: Protocol for the conceptualization and evaluation of a screening-tool for fitness-to-drive assessment in older people with cognitive impairment
Source: PLoS One. 2021 Sep 1;16(9):e0256262. doi: 10.1371/journal.pone.0256262 (PMC8409688; doi:10.1371/journal.pone.0256262)
Supplement: S1 File — (DOCX) [file pone.0256262.s004.docx]

**Studienprotokoll nicht AMG/ nicht MPG**

**Zusammenfassung**

Vor dem Hintergrund des demographischen Wandels in unserer Gesellschaft, nimmt auch die Anzahl von Senioren im Straßenverkehr zu. Eine Vielzahl an altersassoziierten Krankheiten wie demenzielle Syndrome, kardiovaskuläre Erkrankungen oder Muskel-Skelett-Erkrankungen haben Beeinträchtigungen in fahrtauglichkeitsrelevanten motorischen, aber auch kognitiven Leistungen wie Aufmerksamkeit, räumlich-visuelle Wahrnehmung, Reaktionsgeschwindigkeit, Entscheidungsfähigkeit, und Gedächtnis zur Folge^1-3^. Studien konnten zeigen, dass mit fortschreitenden Einbußen in den genannten Leistungsbereichen die Gefahr von Fahrfehlern und die Unfallhäufigkeit im Straßenverkehr steigt^4,5^. Die Untersuchung und Beratung von Patienten bezüglich der Fahreignung ist oftmals mit großen Unsicherheiten verbunden; schlussendlich auch mangels geeigneter, zeitökonomischer Untersuchungsverfahren^6,7^. Entsprechend groß ist der Wunsch nach validen Untersuchungsinstrumenten zur Beurteilung der Fahreignung.

In Zusammenarbeit mit der Firma Schuhfried (GmbH) in Mödling/Wien und der Neuropsychologischen Abteilung des kbo-Inn-Salzach-Klinikums in Wasserburg am Inn, soll ein ökonomisches, für Praktiker im klinischen Alltag problemlos und schnell einsetzbares Fahreignungs-Screening konzipiert werden, dass anhand einer Fahrverhaltensbeobachtung im Realverkehr validiert werden soll. Zu diesem Zweck sollen 40 gesunde Probanden und 40 Probanden mit leichter kognitiver Beeinträchtigung, sowie einem Mindestalter von 50 Jahren, in die Stichprobe eingeschlossen werden.

Zu Beginn werden fahrtauglichkeitsrelevante, kognitive Funktionsbereiche wie Aufmerksamkeit und Exekutivfunktionen mithilfe bereits bewährter, neuropsychologischer Untersuchungsverfahren ausführlich erhoben. Anschließend erfolgt die praktische Fahrverhaltensbeobachtung einem detaillierten Protokoll folgend, in einem Fahrschulwagen, mit einem staatlich anerkannten Fahrlehrer im realen Straßenverkehr. Ergänzt wird die Untersuchung durch eine verkehrsspezifische Anamnese und Fragebögen zur Selbstbeurteilung der Fahreignung. Die Untersuchungen sollten optimalerweise an einem Tag abgeschlossen sein. Anhand der Ergebnisse aus neuropsychologischer Testung und Fahrprobe werden Konstrukt- und Kriteriumsvalidität sowie prädiktiver Wert und Genauigkeit ermittelt.

**1. Studientitel, Versionsnummer, Versionsdatum**

Validierung eines Testsets zur Fahreignungsprüfung von Patienten mit kognitiven Beeinträchtigungen – „DRIVESC2“, Versionsnummer: 1, Versionsdatum: 04.06.2018

**2. Studienleitung, Geldgeber**

Studienleitung: PD Dr. rer. nat. Alexander Brunnauer

Stellvertretende Studienleitung: Prof. Dr. med. Peter Zwanzger

Projektkoordination: Leonhard Zellner, Psychologe (M.Sc.)

Finanzielle Förderung: Firma SCHUHFRIED GmbH, 2340 Mödling, Österreich

**3. Wissenschaftlicher Hintergrund**

Vor dem Hintergrund des demographischen Wandels und der damit einhergehenden steigenden Zahl älterer Menschen in unserer Gesellschaft, nimmt auch die Anzahl von Senioren im Straßenverkehr zu. Eine Vielzahl an altersassoziierten Krankheiten wie demenzielle Syndrome, kardiovaskuläre Erkrankungen oder Muskel-Skelett-Erkrankungen haben Beeinträchtigungen in motorischen und sensorischen, aber auch kognitiven Leistungen zur Folge^1-3,8^, von denen einige, wie z.B. Aufmerksamkeit und Exekutivfunktionen eng mit Fahreignung assoziiert sind^9,10^. Verkehrsteilnehmer müssen visuelle, auditive und taktile Informationen gleichzeitig verarbeiten^1^, adäquat auf diese reagieren, belastbar sein, Distanzen abschätzen, sowie Verkehrszeichen korrekt interpretieren und ihre eigenen Fähigkeiten richtig einschätzen können^8^. Studien konnten zeigen, dass mit Abnahme der kognitiven Funktionalität die Gefahr von Fahrfehlern und die Unfallhäufigkeit im Straßenverkehr zunimmt^4,5^, die Fahreignung dementsprechend abnimmt^12,13^. Vor allem bei dementiellen Erkrankungen, wie beispielsweise einer Alzheimer Erkrankung – der am häufigsten auftretenden Demenzform^14^, die zwangsläufig früher oder später zum Verlust der Fahreignung führt^15,16^ – , die zudem generell einen progressiven Verlauf nimmt, ist das Risiko besonders hoch^17^. Auch die Krankheitseinsicht ist herabgesetzt^18,19^, weshalb eine eigenverantwortliche Überprüfung der Fahreignung im Rahmen der in Deutschland gesetzlich vorgeschriebenen Selbstprüfungs- und Vorsorgepflicht nicht gewährleistet ist^20^. Bereits in frühen Stadien der Demenz ist mit Einschränkungen zu rechnen, Studien weisen darauf hin, dass bereits eine leichte kognitive Beeinträchtigung (MCI; engl. für Mild Cognitive Impairment) mit einer Reduktion der Fahrkompetenz einhergehen kann. MCI beschreibt die Prodromalsymptomatik demenzieller Erkrankungen, die kognitive Beeinträchtigungen beinhaltet, jedoch noch nicht zu einer deutlichen Einschränkung der Alltagskompetenz führt^21^. Ein generelles Fahrverbot oder umfassende Beschränkungen für ältere Menschen mit MCI ist jedoch nicht angebracht, zumal Einbußen in fahrrelevanten Leistungen in frühen Krankheitsstadien noch nicht derartig ausgeprägt sein müssen, um als Rechtfertigung für den Entzug der Fahrerlaubnis zu dienen. Die Begutachtungsleitlinien zur Kraftfahreignung schließen in dieser Phase nicht pauschal die Möglichkeit einer weiteren aktiven Teilnahme am Straßenverkehr aus; jedoch bestehen 13,6% der MCI-Patienten eine praktische Fahrverhaltensprobe oftmals nicht^22^.

In modernen Gesellschaften ist Mobilität ein essentieller Faktor hinsichtlich Lebensqualität und Wohlbefinden. Demzufolge ist der behördliche Entzug der Fahrerlaubnis im Sinne des Schutzes des Patienten und anderer Verkehrsteilnehmer mit den Werten Erhaltung von Selbstbewusstsein, Freiheit, Eigenverantwortlichkeit und Unabhängigkeit zu vergleichen und kritisch abzuwägen^23^. Häufig führt Immobilität zu sozialer Isolation, Inaktivität, Depressionen sowie einer hohen Belastung der betreuenden Angehörigen^24-28^. Daher haben viele ältere Menschen Angst vor dem drohenden Führerscheinverlust, zudem besteht Unkenntnis hinsichtlich der rechtlichen Konsequenzen bei Vernachlässigung der Vorsorgepflicht. Ein möglicher Ansatzpunkt ist eine niederschwellige informelle Mobilitätsberatung, wobei gerade den niedergelassenen Hausärzten, Neurologen, Psychiatern etc. in diesem Bereich ein zentrale Rolle zukommt, sind sie es doch oft, die mit diesem Thema als erste konfrontiert werden und den Patienten hierfür sensibilisieren können.

In der klinischen Praxis ist das Thema Fahreignung mit großen Unsicherheiten verbunden, sowohl auf Seiten der Behandelnden, als auch der Patienten^6,7^. Entsprechend groß ist der Wunsch nach validen Untersuchungsverfahren. Häufig kommen Kurz-Screenings wie der MMST (Mini-Mental-Status-Test) zum Einsatz, die eine grobe Einschätzung des kognitiven Status zulassen, wobei die Studienlage bzgl. des prädiktiven Werts hinsichtlich Fahreignung unbefriedigend ist^29^. Die Ergebnisse neuropsychologischer Tests, speziell in den Bereichen Aufmerksamkeit und visuell-räumliche Wahrnehmung, weisen moderate Korrelationen mit der Performanz im realen Straßenverkehr auf^30,31^. Die als Goldstandard geltende, praxisnahe psychologische Fahrverhaltensbeobachtung kann aufgrund ihrer Ressourcenintensität meist nicht vorgehalten werden.

Generell gilt, dass Einzeltests als Indikatoren für die Fahreignung nicht geeignet sind, da sie kein umfassendes Bild der fahrrelevanten Leistungen abbilden, keinen oder nur einen geringen prädiktiven Wert haben und hohe Sensitivitäts- bzw. Spezifitätswerte nicht erreichen können^30,31^. Einzeltests geben lediglich Hinweise auf mögliche Einschränkungen der Fahreignung, eine praktische Fahrprobe ist dadurch jedoch nicht ersetzbar^32-34^. Bennett & Hons (2016)^35^ stellten fest, dass es keine alleinige kognitive Domäne gibt, die Fahrtüchtigkeit reliabel und valide darstellt^17^. Dies führte zur Schlussfolgerung, dass eine umfassende Testbatterie bestehend aus Testkombinationen und multiplen Testverfahren, welche unterschiedlichste fahrrelevante kognitive Leistungen erhebt, den besten Prädiktor für die Fahreignung bei älteren Personen mit leichter kognitiver Beeinträchtigung darstellt^30,36,37^. Jedoch ist auch hierbei auf die aktuell noch unzureichende Klärung der Validitätskriterien dieser Testbatterien hinzuweisen^38^, welche anhand praktischer Fahrverhaltensbeobachtung und weiterer konstruktnaher und –ferner Verfahren geschehen sollte.

Weiterhin besteht eine große Lücke zwischen wissenschaftlichen Erkenntnissen und klinischer Anwendung; diese wird v.a. durch die fehlenden Cut-Off-Werte der Mehrheit der Forschungsergebnisse erschwert^29^. Nur wenige Studien generierten letztendlich Werte, die im klinischen Alltag eine Unterscheidung zwischen „fahrtauglich“ und „nicht fahrtauglich“ ermöglichen. Aktuelle Empfehlungen beinhalten eine Klassifikationsgenauigkeit von 80-90%^39^, um eine konsistent genaue Vorhersage zu gewährleisten. Darüber hinaus wird im Gegensatz zur bisherigen Dichotomisierung zwischen sicheren und unsicheren Fahrern eine Trichotomisierung vorgeschlagen^29^, was Fahrer in „sicher“, „unklar“ und „unsicher“ einteilen würde, wobei die mittlere Kategorie eine genauere Untersuchung, beispielsweise mithilfe einer Fahrtauglichkeitsbeobachtung, zur exakten Klassifikation nach sich zöge. Bei der Entwicklung dieses Fahrtauglichkeitsscreenings wäre einer effizienten Implementierung in den klinischen Alltag höchste Priorität beizumessen. D.h. sowohl zeitlich, als auch personell dürfte es nicht zu ressourcenbindend sein, was eine computer- oder tabletgestützte Darbietung nahelegt.

Folgende Forschungshypothesen lassen sich ableiten:

1. Kognitiv beeinträchtigte und kognitiv nicht beeinträchtigte Probanden sollten sich signifikant in den Leistungen der Fahrverhaltensbeobachtung unterscheiden
2. Es wird erwartet, dass innerhalb der untersuchten kognitiven Domänen vor allem Funktionstests aus den Bereichen visuell-räumliche Leistungen, Aufmerksamkeit und exekutive Funktionen die Fahrleistung prädizieren
3. Es wird erwartet, dass das neu entwickelte Fahrtauglichkeitsscreening mit hoher diagnostischer Genauigkeit zwischen sicheren und unsicheren Fahrern unterscheiden kann. Die Genauigkeit wird dabei auf mindestens 80% geschätzt und sollte die Genauigkeit von neuropsychologischen Einzeltests deutlich übersteigen.
4. Außerdem wird erwartet, dass die Fahrkompetenz zusätzlich durch Faktoren wie z.B. Alter, Fahrerfahrung in Jahren und Jahreskilometerleistung prädiziert wird.

**4. Studienziele**

Ziel der Studie ist, einen Screening-Test zu entwickeln mit hoher prädiktiver Validität bezüglich der Fahreignung von Personen mit kognitiven Beeinträchtigungen. Konstrukt- und Kriteriumsvalidität sollen mittels standardisierten Fahrprobe sowie konstruktnahen und –fernen Verfahren erfasst werden. Die hohe Praxisrelevanz der Studie ergibt sich aus der Notwendigkeit eines in der Anwendung ökonomischen, validen und reliablen Screenings, tabletgestützt und daher mobil in der klinischen Versorgungspraxis einsetzbar, um eine niedrigschwellige, qualitativ hochwertige und sichere Einschätzung der Fahreignung und Mobilitätsberatung zu unterstützen; schlussendlich auch den gesetzlichen Vorgaben auf Behandler- und Patientenseite gerecht zu werden.

**5. Zielgrößen**

**Primäre Zielgrößen**

Zu den primären Zielgrößen zählen die Leistungen in der neuropsychologischen Untersuchung, speziell in den Bereichen Aufmerksamkeit, visuell-räumliche Fähigkeiten sowie Exekutivfunktionen.

Zudem werden die Leistungen in der Fahrverhaltensbeobachtung – an festgesetzten Beobachtungssequenzen - differenziert bezüglich der Beobachtungskategorien Längsführung, Querführung und Kognition anhand der in diesen Bereichen kodierten Fehler quantifiziert. Jede Beobachtungssequenz wird zudem abschließend auf einer 11-stufigen Skala beurteilt.

**Sekundäre Zielgrößen**

Die aus der verkehrsspezifischen Anamnese erhobenen Daten - soziodemografisch, klinisch und verkehrsspezifisch - werden zusätzlich bezüglich des Einflusses auf die Prädiktion des Fahrverhaltens analysiert.

**6. Studiendesign**

Es handelt sich hierbei um eine nichtrandomisierte Querschnittstudie, die monozentrisch am kbo-Inn-Salzach-Klinikum in Wasserburg durchgeführt wird. Cut-Off-Werte von < 27 und ≥ 18 im MMST weisen die Teilnehmer in die Experimentalgruppe der Probanden mit leichter kognitiver Beeinträchtigung. Probanden, die im MMST Werte von ≥ 27 erreichen, werden der gesunden Kontrollgruppe zugeordnet. Beide Untersuchungsgruppen durchlaufen die gleiche Untersuchungsprozedur. Eine Verblindung ist nur für den Fahrlehrer möglich.

**7. Studienpopulation**

Teilnehmen können Frauen und Männer ab einem Alter von 50 Jahren, die eine gültige Fahrerlaubnis besitzen und aktiv am Straßenverkehr teilnehmen, d.h. regelmäßig in den letzten drei Monaten vor Studienteilnahme gefahren sind, sowie Deutsch in Wort und Schrift sicher beherrschen. Bezüglich einer etwaigen Medikamenteneinnahme ist eine exakte Dokumentation im Rahmen der Anamneseerhebung vorzunehmen und abzuklären, ob sich dadurch eine Beeinträchtigung der Verkehrsteilnahme ergibt. Probanden, die sich in einer medikamentösen Aufdosierungs- bzw. Umstellphase befinden, werden ausgeschlossen. Die Einwilligungsfähigkeit muss überprüft werden, Zweifel daran führen auch zum Ausschluss. Es werden 40 Patienten mit leichter bis mittelgradiger kognitiver Beeinträchtigung und 40 gesunde Kontrollpersonen untersucht, parallelisiert hinsichtlich Alter, Schulbildung, Geschlechtsverteilung, Fahrerfahrung und jährliche Fahrleistung.

*Allgemeine Ausschlusskriterien für alle Teilnehmer (basierend auf Anlage 4, Fahrerlaubnis-verordnung)*

- Eine gegenwärtig schwere Demenzerkrankung (MMST < 18)
- Eine gegenwärtig schwere psychiatrische, neurologische oder internistische Erkrankung
- Visuelle Beeinträchtigungen (zentrale Tagessehschärfe geringer als 60%; Einschränkungen des Gesichtsfeldes (<140˚), Doppelbilder, Halbseiten-Neglekt)
- Kein sicheres Deutsch in Wort und Schrift

*Spezifische Kriterien für die Experimentalgruppen*

Gesunde (KG):

- ≥ 50 Jahre
- Muttersprache Deutsch bzw. deutschsprachig seit frühester Kindheit
- MMST ≥27
- Keine schwere psychiatrische oder neurologische Erkrankung in der Vorgeschichte
- Gegenwärtig keine internistische Erkrankung
- Aktuell keine Strahlen- oder Chemotherapie zur Krebsbehandlung

Kognitiv Beeinträchtigte (EG):

- ≥ 50 Jahre
- Muttersprache Deutsch bzw. deutschsprachig seit frühester Kindheit
- MMST ≥18 und <27
- Patienten aus folgenden ICD-10 Diagnosegruppen
  - Organische, einschließlich symptomatischer psychischer Störungen (F00-F09), N=20
  - Affektive Störungen (F30-F39), N=10
  - Schizophrenie, schizotype und wahnhafte Störungen (F20-F29) / Neurotische, Belastungs- und somatoforme Störungen (F40-F48), N=10

Die Rekrutierung erfolgt per Mail über den Gesamtverteiler an Mitarbeiter des kbo-Inn-Salzach-Klinikums in Wasserburg am Inn, über Aushänge in Mitarbeiterbereichen auf dem Gelände des Klinikums sowie Inserate in einschlägigen Zeitschriften und Informationsbroschüren. Es wird dazu aufgefordert, auch interessierte Freunde und Bekannte auf die Studie aufmerksam zu machen. Zudem werden Patienten über die entsprechenden Abteilungen der Diagnosegruppen F00, F20 und F30 des Inn-Salzach-Klinikums auf die Studie aufmerksam gemacht.

Eine Einschätzung des Schweregrads möglicher psychiatrischer, neurologischer oder internistischer Erkrankungen wird vom behandelnden Arzt/Psychologen des jeweiligen Fachbereichs anhand von Anamnese, Informationen aus der Krankengeschichte sowie vorhandenen Arztbriefen, Befunden etc. vorgenommen. Hinsichtlich der Beurteilung der gesunden Kontrollgruppe muss sich der Testleiter (Psychologe) auf die wahrheitsgemäßen Angaben der Probanden verlassen und anhand deren Angaben eine Einteilung vornehmen

**8. Studienablauf**

Mögliche Teilnehmer werden zunächst schriftlich und mündlich über die Untersuchung aufgeklärt und geben ihr schriftliches Einverständnis.

Die Untersuchung findet möglichst an einem Termin statt. Zu Beginn erfolgt nach einem Vorgespräch, welches die Aufklärung (informed consent) und Anamneseerhebung beinhaltet, die Zuteilung zu Experimental- oder Kontrollgruppe anhand des Ergebnisses im Mini-Mental-Status-Test. Daran schließt sich die neuropsychologische Untersuchung an, die die CFD-Testbatterie (Cognitive Functions Dementia) und den Uhr-Zeichen-Test (CDT) umfasst. Die Dauer der Untersuchung beläuft sich auf ca. 120 Minuten.

Nach einer kurzen Erholungsphase findet die die Fahrverhaltensbeobachtung statt. Zuletzt werden die Ergebnisse der neuropsychologischen Untersuchung und Fahrprobe in einem Nachgespräch rückgemeldet und besprochen sowie mögliche weitere Behandlungsmaßnahmen erläutert.

| **Diagnostik**  Aufklärung  Einverständnis  Anamnese  Mini-Mental-Status-Test  Gruppenzuordnung (nach MMST-Score)  Erfassung Psychopathologie   - Mini-SCL - PDQ   Erfassung der Fahrkompetenz (TQ-Drive)  Neuropsychologische Testdiagnostik   - CFD - Uhr-Zeichen-Test (CDT) - LAT   Dauer: ca. 120 Min. | **Fahrverhaltensbeobachtung**  **& Beratung**  Fahrverhaltensbeobachtung  Ergebnisrückmeldung   - Diagnostik - Fahrverhalten   Informationsmaterial  Dauer: ca. 100 Min. |
| --- | --- |

Abbildung 1: Darstellung des Untersuchungsablaufs

*Aufklärung und Anamnese*

a) Aufklärung und Informationsvermittlung

b) Einverständniserklärung (informed consent)

c) Anamnestisches Gespräch

*Eingesetzte neuropsychologische Untersuchungsverfahren*

| **Test** | **Testform** | **Dauer** | **Anmerkung** |
| --- | --- | --- | --- |
| MMST |  | 10 | CERAD-Plus Version |
| Mini-SCL, PDQ |  | 5 |  |
| Test-Set CFD | S1 | 60 | Standardform plus gekürzte VISCO S3 Form |
| CDT |  | 3 |  |
| LAT | S1 | 10 |  |
| Gesamt |  | 83 |  |

Tabelle 1: Übersicht über die neuropsychologische Untersuchung

a) Mini Mental Status Test (MMST) – Dauer ca. 10 Min.

Der Mini-Mental-Status-Test (Folstein et al., 1975)^40^ ist ein für den klinischen Alltag geeignetes Screening-Verfahren zur Feststellung kognitiver Defizite. Er gilt als zuverlässiges Hilfsmittel zur Erstbeurteilung eines Patienten beispielsweise im Rahmen dementieller Erkrankungen, wie auch zur Verlaufskontrolle. Der Mini-Mental-Status-Test wird als Interview mit dem Patienten durchgeführt. Anhand von 9 Aufgabenkomplexen werden zentrale kognitive Funktionen überprüft (zeitliche und räumliche Orientierung, Merk- und Erinnerungsfähigkeit, Aufmerksamkeit, Sprache und Sprachverständnis, Lesen, Schreiben, Zeichnen und Rechnen). Die Aufgaben des MMST umfassen sowohl das Beantworten von Fragen als auch das Ausführen einfacher Handlungen.

b) Erfassung des subjektiven Beschwerde- bzw. Symptomerlebens

Zur Selbsteinschätzung kognitiver Defizite wird der PDQ (Perceived Deficits Questionnaire; Fehnel et al., 2016)^41^ eingesetzt, anhand dessen Aussagen über die von Patienten erlebte kognitive Beschwerden im Alltag hinsichtlich des Auftretens und der Häufigkeit bewertet werden können. Der Bezugszeitraum umfasst hierbei die letzten sieben Tage.

Die Mini-Symptom-Checklist (Mini-SCL; Franke, 2017)^42^ misst die subjektiv empfundenen Beeinträchtigungen durch vorgegebene körperliche und psychische Symptome im Zeitraum der letzten sieben Tage.

c) Erfassung der Fahrkompetenz (TQ-Drive) – Dauer ca. 7 Min.

Dieser computergestützte Fragebogen erfasst fahranamnestische Informationen, wie Fahrerfahrung bezogen auf Fahrleistung in Kilometer und Jahren, persönliche Bedeutung des Kraftfahrzeugs, Fahrschwierigkeiten und Vermeidungsverhalten etc. Er gibt damit einen Gesamteindruck der individuellen fahrerischen Leistungen und möglicher verkehrsrelevanter Risikofaktoren.

d) Test-Set Kognitive Funktionen Demenz (CFD) – Dauer: ca. 60 Min.

Das Test-Set CFD erfasst neuropsychologische Dimensionen, die für neurokognitive Störungen (laut DSM-5) relevant sind. Mit Hilfe des Test-Sets CFD können kognitive Leistungen aus den neuropsychologischen Dimensionsbereichen Aufmerksamkeit, verbales Langzeitgedächtnis, exekutive Funktionen, expressive Sprache sowie Perzeptuell-motorische Funktionen erfasst werden. In Tabelle 2 sind all jene Einzeltests in der Reihenfolge der Vorgabe inkl. Durchführungsdauer aufgelistet, welche in der Studie zum Einsatz kommen.

|  | Dimension | Test | Testform | Dauer |
| --- | --- | --- | --- | --- |
| 1 | Semantische Wortflüssigkeit | WIWO | S1 | 3 |
| 2 | Lexikalische Wortflüssigkeit | WIWO | S3 | 4 |
| 3 | Verbales Langzeitgedächtnis  Subdimension: Lernfähigkeit | AWLT  (Subtest 1) | S1 | 7 |
| 4 | Alertness (intrinsisch-visuell) | WAFA | S2 | 2 |
| 5 | Verbales Langzeitgedächtnis  Subdimension: Kurz verzögerter Abruf | AWLT  (Subtest 2) | S1 | 2 |
| 6 | Geteilte Aufmerksamkeit | WAFG | S3 | 9 |
| 7 | Verarbeitungsgeschwindigkeit | TMT | S1 | 1 |
| 8 | Kognitive Flexibilität | TMT | S1 | 1 |
| 9 | Arbeitsgedächtnis, räumlich | CORSI | S7 | 8 |
| 10 | Verbales Langzeitgedächtnis  Subdimensionen: Lang verzögerter Abruf und Wiedererkennen | AWLT  (Subtests  3 und 4) | S1 | 4 |
| 11 | Objektbenennung | WOBT | S1 | 5 |
| 12 | Visuokonstruktion | VISCO | S3 | 7 |

Tabelle 2: Dimensionen und Tests des Test-Sets CFD

Die in Tabelle 2 einzeln angeführten Tests werden nun im Detail vorgestellt.

Zu 1. und 2.: Wiener Wortflüssigkeitstest (WIWO)

Die Wortflüssigkeit beschreibt die Fähigkeit einer Person, Wörter entsprechend vorgegebener Regeln zu generieren und wiederzugeben. Dabei können zwei Subdimensionen der Wortflüssigkeit unterschieden werden: die semantische und die lexikalische Wortflüssigkeit. Während die semantische Wortflüssigkeit die Fähigkeit einer Person beschreibt, Wörter einer bestimmten Kategorie wiederzugeben (z. B. Vornamen), erfasst die lexikalische Wortflüssigkeit die Fähigkeit, Wörter mit bestimmten Anfangsbuchstaben zu generieren.

Im Test-Set CFD werden die beiden Subdimensionen der Wortflüssigkeit mit dem Verfahren WIWO erfasst. Die Aufgabe der Testpersonen ist es, innerhalb von zwei Minuten möglichst viele Wörter zu nennen, die einer bestimmten Kategorie angehören (semantische Wortflüssigkeit: Testformen S1) oder mit einem bestimmten Buchstaben beginnen (lexikalische Wortflüssigkeit: Testformen S2). Als Hauptvariable wird die Anzahl der richtig genannten Wörter herangezogen.

Zu 3., 5. und 10.: Auditiver Wortlisten Lerntest (AWLT)

Das Langzeitgedächtnis beschreibt die Fähigkeit Informationen längerfristig zu behalten, in die eigene Wissensbasis zu integrieren und bei Bedarf abzurufen. Das Langzeitgedächtnis enthält alle Informationen, die nach einer Zeitspanne über wenige Minuten hinaus noch erinnert werden können. Im Prozess des Lernens sind die Phasen der Enkodierung, der Konsolidierung und des Abrufs zu unterschieden.

Das verbale Langzeitgedächtnis wird im Test-Set CFD mit dem Verfahren AWLT mit der Testform S1 erfasst. Als Lernmaterial wird im AWLT eine Liste mit 12 Wörtern verwendet. Die Messung erfolgt durch Umsetzung einer Lern-Prüf-Methode mit vier Lerndurchgängen sowie einem kurz (5 Minuten Pause) und einem lang (20 Minuten Pause) verzögerten freien Abruf. Unmittelbar nach jedem Lerndurchgang sowie beim kurz und lang verzögerten Abruf müssen so viele Wörter wie möglich frei reproduziert werden. Bei den beiden Abrufphasen wird die Wortliste nicht noch einmal dargeboten. Zum Abschluss wird eine Liste mit 24 Wörtern präsentiert: 12 Wörter der Liste aus der Lernphase und 12 neue Wörter als Distraktoren. Aufgabe der Testperson ist es anzugeben, welche der 24 Wörter in der Wortliste der Lernphase enthalten waren und welche nicht. Als Hauptvariablen werden die Maße „Lernsumme“, „Kurz verzögerter Abruf“, „Lang verzögerter Abruf“ und „Wiedererkennen“ herangezogen.

Zu 4.: Wahrnehmungs- und Aufmerksamkeitsfunktionen – Alertnes (WAFA)

Alertness zählt zum übergeordneten Dimensionsbereich „Aufmerksamkeit“. Das Konstrukt der Alertness beschreibt einerseits den Zustand anhaltender allgemeiner Wachheit und Reaktionsbereitschaft (tonische Alertness) und andererseits die Fähigkeit, das Aufmerksam­keitsniveau nach einem Warnreiz kurzfristig zu steigern (phasische Alertness). Erfolgt die Reizdarbietung ohne Warnreiz, so wird auch von „intrinsischer Alertness“ gesprochen. Im Gegensatz zur phasischen Alertness, kommt es bei der intrinsischen Alertness zu einer selbstgenerierten Steigerung des Aktivierungsniveaus.

Alertness wird im Test-Set CFD mit dem Verfahren WAFA mit der Testform S2 erfasst. Im Test WAFA wird die Reaktionszeit auf einfaches visuelles Reizmaterial (schwarzer Kreis auf weißem Hintergrund) gemessen. Als Hauptvariable wird die „Mittlere Reaktionszeit“ verwendet.

Zu 6.: Wahrnehmungs- und Aufmerksamkeitsfunktionen – Geteilte Aufmerksamkeit (WAFG):

Geteilte Aufmerksamkeit zählt zum übergeordneten Dimensionsbereich „Aufmerksamkeit“. Geteilte Aufmerksamkeit bezeichnet die Fähigkeit, die Aufmerksamkeit auf mehrere Informationskanäle zugleich zu richten. Die Teilung der Aufmerksamkeit wird beispielsweise in „Dual-Task-Aufgaben“ verlangt, in denen zwei Informationskanäle simultan überwacht werden müssen. Tritt in einem oder in beiden Kanälen ein relevantes Ereignis ein, muss so schnell wie möglich reagiert werden.

Die Erfassung der geteilten Aufmerksamkeit erfolgt im Test-Set CFD mit dem Verfahren WAFG, mit der Testform S3. Die Testperson erhält Reizmaterial auf einem visuellen und einem auditiven Kanal. Sie soll dabei stets beide Kanäle dahingehend überwachen, ob einer der Zielreize (Quadrat bzw. hoher Ton) zweimal hintereinander dargeboten wird. Als Hauptvariable wird die „Mittlere Reaktionszeit“ herangezogen.

Zu 7.: Trail Making Test – Langensteinbacher Version (TMT-L) – Teil A:

Verarbeitungsgeschwindigkeit ist die basale Fähigkeit, einfache Reize schnell und sicher zu bearbeiten, ohne dass hierfür die Beteiligung höherer kognitiver, sensorischer oder motorischer Prozesse ausschlaggebend ist. Der Teil A des Verfahrens TMT-L in den Testformen S1 dient als grundlegendes Maß für die Verarbeitungsgeschwindigkeit. Das Aufgabenmaterial besteht aus mehreren Kreisen, welche gleichzeitig am Bildschirm gezeigt werden und die Zahlen von 1 bis 25 enthalten. Diese sind möglichst schnell in aufsteigender Reihenfolge zu verbinden. Als Hauptvariable wird die „Bearbeitungszeit Teil A“ herangezogen.

Zu 8.: Trail Making Test – Langensteinbacher Version (TMT) – Teil B:

Im Teil B des TMT wird hingegen die kognitive Flexibilität mit der Testform S1 erfasst. Kognitive Flexibilität bezeichnet die Fähigkeit, flexibel zwischen unterschiedlichen Bezugssystemen wechseln zu können, sofern dies gefordert ist. Die Aufgabe der Testperson besteht darin, alternierend Kreise mit den Zahlen 1 bis 13 bzw. den Buchstaben A bis L in jeweils aufsteigender Reihenfolge zu verbinden. Als Hauptvariable wird die „Bearbeitungszeit im Teil B“ verwendet.

Zu 9.: Corsi-Block-Tapping-Test (CORSI):

Räumliches Arbeitsgedächtnis zählt zum übergeordneten Dimensionsbereich „Exekutive Funktionen“. Das Arbeitsgedächtnis umfasst die Fähigkeit, Gedächtnisinhalte in Gedanken zu bearbeiten, um Aufgaben zu erfüllen und Ziele zu erreichen. Unter Arbeitsgedächtnis kann daher die Fähigkeit zur Aufrechterhaltung und Bearbeitung kurzfristig dargebotener Informationen verstanden werden, die für die Lösung einer aktuell anstehenden Aufgabe relevant sind.

Die Erfassung des (räumlichen) Arbeitsgedächtnisses erfolgt im Test-Set CFD mit dem Verfahren CORSI in der Testform S7. Dabei werden auf dem Bildschirm neun Blöcke präsentiert. Bei jeder Aufgabe wird von einem sich über den Bildschirm bewegenden Handsymbol eine Reihe von Würfeln angetippt. Diese sind von der Testperson in der umgekehrten Reihenfolge (rückwärts) anzutippen. Die Länge der Sequenzen nimmt dabei im Testverlauf zu. Die Vorgabe beginnt mit einer 2er-Sequenz und endet spätestens bei der 9er-Sequenz. Der Test bricht ab, sobald drei Sequenzen in Folge falsch angetippt wurden. Als Hauptvariable wird die Variable „Unmittelbare Blockspanne rückwärts“ herangezogen.

Zu 11.: Wiener Objektbenennungstest (WOBT):

Objektbenennung zählt zum übergeordneten Dimensionsbereich „Expressive Sprache“. Objektbenennung beschreibt die Fähigkeit, vorgezeigte Objekte zu erkennen und die entsprechenden Bezeichnungen aus dem Wortschatz abzurufen und wiederzugeben. Insofern ist an der Testleistung auch das deklarative Altgedächtnis beteiligt. Objektbenennung wird im Test-Set CFD mit dem Verfahren WOBT in der Testform S1 erfasst. Im Test werden der Testperson Bilder mit Abbildungen von unterschiedlichen Objekten gezeigt und die Aufgabe der Person ist es, diese Objekte richtig zu benennen. Gelingt der Testperson die Objektbenennung nicht, wird zunächst ein lexikalischer Hinweis (Anfangsbuchstabe des Zielobjektnamens) und gegebenenfalls in einem weiteren Schritt auch ein semantischer Hinweis (inhaltliche Beschreibung des Zielobjekts ohne das Zielwort zu nennen) gegeben. Hilft der Testperson keiner dieser Hinweise das Objekt zu benennen, wird mit dem nächsten Item weitergemacht. Als Hauptvariable wird die Anzahl der unmittelbar richtigen Benennungen („Richtige Benennungen unmittelbar“) herangezogen.

Zu 12.: Visuokonstruktionstest (VISCO):

Visuokonstruktionsfähigkeit zählt zum übergeordneten Dimensionsbereich „Perzeptuell-motorische Funktionen“. Visuokonstruktion beschreibt die Fähigkeit einzelne Elemente von Formen oder Objekten zu erkennen und diese Formen oder Objekte durch Zusammensetzen der einzelnen Elemente zu (re)konstruieren.

Diese Fähigkeit wird im Test-Set CFD mit dem Verfahren VISCO in der Testform S3 erfasst. Der Test besteht aus 16 Aufgaben, wobei bei jeder Aufgabe eine aus mehreren gleichseitigen Dreiecken zusammengesetzte Form als Zielfigur dargeboten wird. Hierbei ist nur der Gesamtumriss der Figur zu sehen, nicht die Umrisse der einzelnen Dreiecke selbst. Aufgabe der Testperson ist es, den Aufbau der Zielfigur nachzuvollziehen und sie in einem Eingabefeld mithilfe von nach oben und nach unten gerichteten Dreiecken selbst zu konstruieren. Für die Bearbeitung einer Aufgabe stehen maximal 60 Sekunden zur Verfügung. Sobald es einer Person bei drei Aufgaben in Folge nicht gelingt, die richtige Figur zusammen zu setzen, kommt es automatisch zum Abbruch des Tests. Als Hauptvariable wird die Variable „Visuokonstruktionsfähigkeit“ verwendet.

e) Uhr-Zeichen-Test (clock drawing test; CDT)

Beim Uhren-Zeichen-Test nach Shulman (1993)^43^ handelt es sich um ein weitverbreitetes Schnell-Screening zur Prüfung visuokonstruktiver Fähigkeiten zum Beispiel während der ersten diagnostischen Abklärung dementieller Erkrankungen. Der Patient wird gebeten, das Ziffernblatt einer Uhr zu zeichnen und die Zeigerstellung einer bestimmten Uhrzeit einzutragen. Die Durchführung dauert ca. drei Minuten. Anhand der Abweichungen in der Darstellung von der "Normal-Leistung" (Aufteilung des Zifferblattes, Schriftbild der Zahlen, Fehlen der Zeiger und Ähnliches) lassen sich Rückschlüsse auf das Ausmaß der Funktionsstörung ziehen. Dabei werden je nach Abweichung 1 bis 6 Punkte vergeben, wobei ein Score ≥ 3 als Hinweis auf Funktionsbeeinträchtigungen in diesem Bereich gewertet werden kann.

f) Linien-Ausricht-Test (LAT)

Die Raumverarbeitungsprozesse des Gehirns werden üblicherweise in vier Kategorien eingeteilt: räumlich-perzeptive, räumlich-kognitive, räumlich-konstruktive und räumlich-topografische Leistungen. Räumlich-perzeptive Leistungen sind vergleichsweise elementarer Art. Zu ihnen gehören die Lagebestimmung der subjektiven Hauptraum-achsen (Vertikale, Horizontale), die Orientierung (Neigungswinkel), die Erfassung des Raumes innerhalb von Objekten (Länge, Größe) wie auch zwischen Objekten (Distanzen), das Erkennen von Positionen und Konturen, die Formschätzung sowie speziellere Leistungen wie die Linienhalbierung und die subjektive Geradeausrichtung.

LAT erfasst basale räumlich-perzeptive Fähigkeiten, insbesondere die Fähigkeit Neigung von Linien zu beurteilen und Winkel zwischen Linien zu schätzen. Beim LAT werden auf einer Bildschirmseite zwei Pfeile abgebildet, die sich an der Basis berühren und in einem bestimmten Winkel zueinanderstehen. Auf der anderen Bildschirmseite ist ein Ziffernblatt. Am Ziffernblatt müssen jene zwei Zahlen markiert werden, auf welche die Pfeile zeigen würden, wenn sie sich in der Mitte des Zifferblattes befinden. Als Hauptvariable wird die Anzahl der richtig bearbeiteten Aufgaben verwendet.

g) Fahrverhaltensbeobachtung

Die Wasserburger Fahrverhaltensbeobachtung (WAFAB) wird von einem staatlich geprüften, in der Beurteilung von Kraftfahrern mit neurologischen oder psychischen Erkrankungen erfahrenen Fahrlehrer und einer Assistentin in einem standardisierten Fahrschulfahrzeug (Pkw) durchgeführt. Sie umfasst eine etwa 50-minütige Fahrt entlang einer festgelegten Route von 50 Kilometer Länge, mit definierten Beobachtungspunkten, die in der klinischen Praxis in den letzten zehn Jahren erprobt wurde und sich bewährt hat. Der Fahrlehrer ist bezüglich der Gruppenzuteilung nicht informiert. Als globales Maß wird jede Beobachtungssequenz auf einer 11-stufigen Fitness-to-Drive-Skala^44^ beurteilt. Die Skala besteht aus drei verbalen Kategorien (normal, eingeschränkt, kritisch) sowie drei numerischen Subkategorien (hoch, mittel, niedrig). Die Fahrfehler werden zudem von geschulten Ratern während der Fahrt hinsichtlich Gesamtfehlerzahl, Längsregelungsfehler (Geschwindigkeitsverhalten, Längsabstand), Querregelungsfehler (Abkommen von der Fahrbahn, Querabstand) und kognitiven Fehlern (Spurwechsel, Fahrbahnbenutzung, Sicherungsverhalten, Kommunikation, Navigationsfehler) an festgelegten Beobachtungspunkten bewertet; als Zusatzkategorie werden zudem kritische Situationen (Gefährdungen, Kollisionen) erfasst.

**9. Nutzen-Risiko-Abwägung**

Die Probanden befinden sich zum Untersuchungszeitpunkt in einer Art Prüfungssituation. Dies kann für sie sich als belastend herausstellen, möglicherweise werden unangenehme Gefühle ausgelöst. Jedoch findet eine ausführliche Aufklärung über mögliche Belastungsfaktoren statt. Zu jedem Zeitpunkt der Untersuchung ist ein direkter Kontakt zu psychologisch geschultem Untersuchungspersonal gegeben, was ein schnelles Mitteilen von Beschwerden und sofortiges Reagieren darauf garantiert. Zudem kann die Untersuchung jederzeit abgebrochen werden. Die Auftretenswahrscheinlichkeit weiterer Risiken wird als sehr niedrig eingeschätzt. Eine Inanspruchnahme von Pausen ist zu jedem Zeitpunkt erlaubt.

Die Probanden erhalten im Rahmen der Studienteilnahme eine kostenlose und professionelle Einschätzung Ihrer Fahrtauglichkeit sowie eine Aufwandsentschädigung in Höhe von 25 Euro.

Für die Etablierung eines neuen Fahrtauglichkeitsscreenings ist es von grundlegender Bedeutung, die prädiktive Validität sowie Genauigkeit des Verfahrens zu kennen. Wir schätzen das Nutzen-Risiko-Verhältnis als günstig ein, da die Risiken sehr gering, der Nutzen eines ökonomischen, prädiktiv wertvollen Fahrtauglichkeits-screenings für den klinischen Alltag als hoch zu werten ist.

**10. Biometrie**

Studien mit neurologischen Patienten zum Zusammenhang zwischen Fahrverhalten und kognitiven Tests zeigen eine mittlere bis große Effektstärken für TMT (Teil A und B) und visuell räumlichen Tests wie den Judgement of Line Orientation Test^16,17,22,45^. Demnach wird davon ausgegangen, dass auch TMT-L (Teil A und B) und LAT das Fahrverhalten mit einer mittleren Effektstärke vorhersagen können. Die geplante Stichprobengröße von N = 80 ergibt sich demnach wie folgt: Bei einer Power (1 – β) von 80% und einem Alphaniveau von α = .05 ist eine Stichprobengröße von insgesamt N = 77 erforderlich um einen mittleren Effekt bei einer multiplen Regression mit drei Prädiktoren (TMT-L Teil A, Teil B und LAT) zu detektieren^46^. Für den Fall vorzeitiger Abbrüche kalkulieren wir eine Stichprobengröße von N=80 zur Rekrutierung.

Die Daten werden mittels multivariater Varianzanalysen mit anschließenden post-hoc-t-Tests sowie Regressionsanalysen zur Feststellung der prädiktiven Validität der Einzeltests ausgewertet.

Die Voraussetzungen der statistischen Berechnungen, wie Normalverteilung der Stichproben oder Varianzgleichheit, sind im Vorfeld zu überprüfen.

**11. Datenmanagement**

Alle erhobenen Daten dienen ausschließlich Forschungszwecken, werden streng vertraulich behandelt und nach der Erhebung irreversibel anonymisiert sowie für unbestimmte Zeit gespeichert. Die Studienergebnisse werden nach Abschluss der Studie wissenschaftlich ausgewertet und in anonymisierter Form, die keinen Rückschluss auf die Person des Probanden zulässt, veröffentlicht.

**12. Versicherung**

Die Studie ist im Rahmen der bestehenden Betriebshaftpflichtversicherung sowie der fahrschuleigenen Versicherung versichert.

**13. Unterschrift**

________________________________________________________

Wasserburg am Inn, den

**14. Literaturverzeichnis**

1. Anstey, K. J., Wood, J., Lord, S., & Walker, J. G. (2005). Cognitive, sensory and physical factors enabling driving safety in older adults. *Clinical psychology review*, *25*(1), 45-65.

2. McKhann, G. M., Knopman, D. S., Chertkow, H., Hyman, B. T., Jack, C. R., Kawas, C. H., ... & Mohs, R. C. (2011). The diagnosis of dementia due to Alzheimer’s disease: Recommendations from the National Institute on Aging-Alzheimer’s Association workgroups on diagnostic guidelines for Alzheimer's disease. *Alzheimer's & dementia: the journal of the Alzheimer's Association*, *7*(3), 263-269.

3. Smits, L. L., Pijnenburg, Y. A., van der Vlies, A. E., Koedam, E. L., Bouwman, F. H., Reuling, I. E., ... & van der Flier, W. M. (2015). Early onset APOE E4-negative Alzheimer’s disease patients show faster cognitive decline on non-memory domains. *European Neuropsychopharmacology*, *25*(7), 1010-1017.

4. Evans, L. (2004). *Traffic safety*.

5. McKnight, A. J., & McKnight, A. S. (1999). Multivariate analysis of age-related driver ability and performance deficits. *Accident Analysis & Prevention*, *31*(5), 445-454.

6. Jones, K., Rouse-Watson, S., Beveridge, A., Sims, J., & Schattner, P. (2012). Fitness to drive: GP perspectives of assessing older and functionally impaired patients. *Australian family physician*, *41*(4), 235.

7. Jang, R. W., Man-Son-Hing, M., Molnar, F. J., Hogan, D. B., Marshall, S. C., Auger, J., ... & Naglie, G. (2007). Family physicians’ attitudes and practices regarding assessments of medical fitness to drive in older persons. *Journal of general internal medicine*, *22*(4), 531-543.

8. Hedden, T., & Gabrieli, J. D. (2004). Insights into the ageing mind: a view from cognitive neuroscience. *Nature reviews neuroscience*, *5*(2), 87.

9. Golz, D., Huchler, S., Jörg, A., & Küst, J. (2004). Beurteilung der Fahreignung. *Zeitschrift für Neuropsychologie*, *15*(3), 157-167.

10. Falkenstein, M., & Sommer, S. M. (2008). Altersbegleitende Veränderungen kognitiver und neuronaler Prozesse mit Bedeutung für das Autofahren. *Prof. Dr.-Ing. Bernd H. Müller Forschungsstelle Mensch-Verkehr der Eugen-Otto-Butz-Stiftung*, 1885.

11. Lundberg, C., Johansson, K., Ball, K., Bjerre, B., Blomqvist, C., Braekhus, A., ... & Friedland, R. P. (1997). Dementia and driving: an attempt at consensus. *Alzheimer disease and associated disorders*, *11*(1), 28-37.

12. Anstey, K. J., & Wood, J. (2011). Chronological age and age-related cognitive deficits are associated with an increase in multiple types of driving errors in late life. *Neuropsychology*, *25*(5), 613.

13. Kroll, G., Kaiser, A., Krone, M., Mönning, M., Griese, H., Macek, C. E. E. A., & Hartje, W. (2003). Die praktische Fahrprobe im mittleren und höheren Lebensalter. *Zeitschrift für Neuropsychologie*, *14*(2), 81-87.

14. Deuschl, G., & Meier, W. et al. *S3-Leitlinie Demenzen. 2016.* In: Deutsche Gesellschaft für Neurologie, Hrsg. Leitlinien für Diagnostik und Therapie in der Neurologie. Online: [www.dgn.org/leitlinien](http://www.dgn.org/leitlinien) [22.11.2016]

15. Duchek, J. M., Carr, D. B., Hunt, L., Roe, C. M., Xiong, C., Shah, K., & Morris, J. C. (2003). Longitudinal driving performance in early‐stage dementia of the Alzheimer type. *Journal of the American Geriatrics Society*, *51*(10), 1342-1347.

16. Ott, B. R., Heindel, W. C., Papandonatos, G. D., Festa, E. K., Davis, J. D., Daiello, L. A., & Morris, J. C. (2008). A longitudinal study of drivers with Alzheimer disease. *Neurology*, *70*(14), 1171-1178.

17. Reger, M. A., Welsh, R. K., Watson, G., Cholerton, B., Baker, L. D., & Craft, S. (2004). The relationship between neuropsychological functioning and driving ability in dementia: a meta-analysis. *Neuropsychology*, *18*(1), 85.

18. Kessler, H., & Supprian, T. (2003). Zum Problem der Krankheitseinsicht bei Patienten mit Demenz vom Alzheimer-Typ.

19. Vogel, A., Stokholm, J., Gade, A., Andersen, B. B., Hejl, A. M., & Waldemar, G. (2004). Awareness of deficits in mild cognitive impairment and Alzheimer’s disease: Do MCI patients have impaired insight?. *Dementia and geriatric cognitive disorders*, *17*(3), 181-187.

20. Neumann-Zielke, L. (2004). Die Position Klinischer Neuropsychologen in der Rehabilitation von Kraftfahrern. *Zeitschrift für Neuropsychologie*, *15*(3), 189-207.

21. Albert, M. S., DeKosky, S. T., Dickson, D., Dubois, B., Feldman, H. H., Fox, N. C., ... & Snyder, P. J. (2011). The diagnosis of mild cognitive impairment due to Alzheimer’s disease: Recommendations from the National Institute on Aging-Alzheimer’s Association workgroups on diagnostic guidelines for Alzheimer's disease. *Alzheimer's & dementia: the journal of the Alzheimer's Association*, *7*(3), 270-279.

22. Hird, M. A., Egeto, P., Fischer, C. E., Naglie, G., & Schweizer, T. A. (2016). A systematic review and meta-analysis of on-road simulator and cognitive driving assessment in Alzheimer’s disease and mild cognitive impairment. *Journal of Alzheimer's disease*, *53*(2), 713-729.

23. Post, S. G. (2000). Key issues in the ethics of dementia care. *Neurologic clinics*, *18*(4), 1011-1022.

24. Fonda, S. J., Wallace, R. B., & Herzog, A. R. (2001). Changes in driving patterns and worsening depressive symptoms among older adults. *The Journals of Gerontology Series B: Psychological Sciences and Social Sciences*, *56*(6), S343-S351.

25. Marottoli, R. A., Leon, C. F. M., Glass, T. A., Williams, C. S., Cooney, L. M., Berkman, L. F., & Tinetti, M. E. (1997). Driving cessation and increased depressive symptoms: Prospective evidence from the New Haven EPESE. *Journal of the American Geriatrics Society*, *45*(2), 202-206.

26. Marottoli, R. A., de Leon, C. F. M., Glass, T. A., Williams, C. S., Cooney Jr, L. M., & Berkman, L. F. (2000). Consequences of driving cessation: decreased out-of-home activity levels. *The Journals of Gerontology Series B: Psychological Sciences and Social Sciences*, *55*(6), S334-S340.

27. Ragland, D. R., Satariano, W. A., & MacLeod, K. E. (2005). Driving cessation and increased depressive symptoms. *The Journals of Gerontology Series A: Biological Sciences and Medical Sciences*, *60*(3), 399-403.

28. Windsor, T. D., & Anstey, K. J. (2006). Interventions to reduce the adverse psychosocial impact of driving cessation on older adults. *Clinical interventions in aging*, *1*(3), 205.

29. Molnar, F. J., Patel, A., Marshall, S. C., Man‐Son‐Hing, M., & Wilson, K. G. (2006). Clinical Utility of Office‐Based Cognitive Predictors of Fitness to Drive in Persons with Dementia: A Systematic Review. *Journal of the American Geriatrics Society*, *54*(12), 1809-1824.

30. Dawson, J. D., Anderson, S. W., Uc, E. Y., Dastrup, E., & Rizzo, M. (2009). Predictors of driving safety in early Alzheimer disease. *Neurology*, *72*(6), 521-527.

31. Anderson, S. W., Aksan, N., Dawson, J. D., Uc, E. Y., Johnson, A. M., & Rizzo, M. (2012). Neuropsychological assessment of driving safety risk in older adults with and without neurologic disease. *Journal of clinical and experimental neuropsychology*, *34*(9), 895-905.

32. Brunnauer, A., Buschert, V., & Laux, G. (2014). Demenz und Autofahren. *Der Nervenarzt*, *85*(7), 811-815.

33. Iverson, D. J., Gronseth, G. S., Reger, M. A., Classen, S., Dubinsky, R. M., & Rizzo, M. (2010). Practice parameter update: Evaluation and management of driving risk in dementia report of the Quality Standards Subcommittee of the American Academy of Neurology. *Neurology*, *74*(16), 1316-1324.

34. Poschadel, S., Falkenstein, M., Pappachan, P., Poll, E., & Willmes von Hinckeldey, K. (2009). Testverfahren zur psychometrischen Leistungsprüfung der Fahreignung. *BERICHTE DER BUNDESANSTALT FUER STRASSENWESEN. UNTERREIHE MENSCH UND SICHERHEIT*, (203).

35. Bennett, J. M., Chekaluk, E., & Batchelor, J. (2016). Cognitive tests and determining fitness to drive in dementia: a systematic review. *Journal of the American Geriatrics Society*, *64*(9), 1904-1917.

36. Dawson, J. D., Uc, E. Y., Anderson, S. W., Johnson, A. M., & Rizzo, M. (2010). Neuropsychological predictors of driving errors in older adults. *Journal of the American Geriatrics Society*, *58*(6), 1090-1096.

37. Wood, J. M., Anstey, K. J., Kerr, G. K., Lacherez, P. F., & Lord, S. (2008). A multidomain approach for predicting older driver safety under in‐traffic road conditions. *Journal of the American Geriatrics Society*, *56*(6), 986-993.

38. Bowers, A. R., Anastasio, R. J., Sheldon, S. S., O’Connor, M. G., Hollis, A. M., Howe, P. D., & Horowitz, T. S. (2013). Can we improve clinical prediction of at-risk older drivers?. *Accident Analysis & Prevention*, *59*, 537-547.

39. Bedard, M., Weaver, B., Dārzin, P., & Porter, M. M. (2008). Predicting driving performance in older adults: we are not there yet!. *Traffic injury prevention*, *9*(4), 336-341.

40. Folstein, M. F., Folstein, S. E., & McHugh, P. R. (1975). “Mini-mental state”: a practical method for grading the cognitive state of patients for the clinician. *Journal of psychiatric research*, *12*(3), 189-198.

41. Franke, G. H. (2017). *Mini-Syptom-Checkliste.* Hogrefe.

42. Fehnel, S. E., Forsyth, B. H., DiBenedetti, B. D., Danchenko, N., François, C., & Brevig, T. (2016). Patient centered assessment of cognitive symptoms of depression*.* [*CNS Spect,*](https://www.ncbi.nlm.nih.gov/pubmed/24067243)*21(1)*, 43-52.

43. Shulman, K. I., Pushkar Gold, D., Cohen, C. A., & Zucchero, C. A. (1993). Clock‐drawing and dementia in the community: A longitudinal study. *International journal of geriatric psychiatry*, *8*(6), 487-496.

44. Neukum, A., & Krüger, H. P. (2003). Fahrerreaktionen bei Lenksystemstörungen–Untersuchungsmethodik und Bewertungskriterien. *VDI-Berichte*, *1791*, 297-318.

45. Grace, J., Amick, M. M., D'abreu, A., Festa, E. K., Heindel, W. C., & Ott, B. R. (2005). Neuropsychological deficits associated with driving performance in Parkinson's and Alzheimer's disease. *Journal of the International Neuropsychological Society*, *11*(6), 766-775.

46. Faul, F., Erdfelder, E., Lang, A. G., & Buchner, A. (2007). G* Power 3: A flexible statistical power analysis program for the social, behavioral, and biomedical sciences. *Behavior research methods*, *39*(2), 175-191.
