# Supplementary material for: Protocol for the conceptualization and evaluation of a screening-tool for fitness-to-drive assessment in older people with cognitive impairment
Source: PLoS One. 2021 Sep 1;16(9):e0256262. doi: 10.1371/journal.pone.0256262 (PMC8409688; doi:10.1371/journal.pone.0256262)
Supplement: S2 File — (DOCX) [file pone.0256262.s005.docx]

**Study protocol not AMG/ not MPG**

**Summary**

In the light of demographic change the number of elders in road traffic increases. A variety of age related illnesses like dementia, cardiovascular or musculoskeletal diseases lead to impairments in driving related motoric but also cognitive functions like attention, visuospatial perception, reaction speed, decision-making ability and memory^1-3^. Studies could show that with increasing loss in functions mentioned above the risk of driving errors and accident frequency in road traffic also rises^4,5^. The assessment and counselling of patients in clinical practice with regard to driving safety is often associated with uncertainties also due to a lack of appropriate assessment tools^6,7^. Hence there is a distinct necessity of valid assessment instruments examining driving safety in elder patients.

In cooperation with Schuhfried (GmbH) from Mödling/Vienna and the Neuropsychological Department of the kbo-Inn-Salzach-Klinikum in Wasserburg/Inn, an economical, in clinical routine practice easily applicable assessment tool for driving safety will be conceptualized and validated on the basis of an on-road driving observation. For this purpose a sample of 40 healthy subjects and 40 subjects with mild cognitive impairment, all with a minimum age of 50 years, will be targeted.

At the beginning driving related cognitive functions like attention and executive functions will be comprehensively examined by means of established neuropsychological assessment instruments. Subsequently the on-road driving observation will be carried out, following a detailed protocol, in a driving instruction vehicle, with a licensed driving instructor. The assessment will be supplemented by driving specific anamnesis and questionnaires for self-assessment of driving safety. It should optimally be finished within one day. Construct validity, criterion-related validity as well as predictive value will be determined by means of the results from neuropsychological examination and the on-road assessment.

**1 Study title, version number, version date**Validation of a test set for assessment of driving safety of patients with cognitive impairment – „DRIVESC2“, version number: 1, version date: 06/04/2018


**2. Study direction, foundation**Principal investigator: PD Dr. rer. nat. Alexander Brunnauer

Deputy principal investigator: Prof. Dr. med. Peter Zwanzger

Project coordination: Leonhard Zellner, Psychologist (M.Sc.)

Foundation: SCHUHFRIED GmbH, 2340 Mödling, Austria


**3. Scientific background**

As a consequence of demographic change the number of elders in road traffic also increases. A variety of age related illnesses like dementia, cardiovascular or musculoskeletal diseases lead to impairments in motoric, sensorial and cognitive functions^1-3,8^, some of which, like attention and executive function, are closely related to driving safety^9,10^. Traffic participants have to process visual, acoustic and tactile information simultaneously, react adequately to them, be stressable, estimate distances as well as correctly interpret traffic signs and judge their own driving abilities properly^8^. Studies could show that with increasing loss of cognitive functionality the risk of driving errors and accident frequency in road traffic rises^4,5^, the driving safety as a consequence decreases^12,13^. Especially in dementia, for example Alzheimer’s disease – the most common form of dementia^14^, which inevitably sooner or later leads to a loss of driving ability^15,16^ – and furthermore has a progressive process, the risk is particularly high^17^. Also the insight into illness is degraded^18,19^, which is why the self-reliant examination of one’s own driving ability in the context of duty to precaution and self review required by law in Germany is not guaranteed^20^. Already in early stages of dementia impairment should be taken into account. Studies point out that even mild cognitive impairment (MCI) can be accompanied by a decrease of driving competence. MCI describes the prodromal symptoms of dementia diseases, which comprises cognitive impairments, however does not lead to notable constraints in functioning independently. A general driving ban or broad restrictions for seniors with MCI nevertheless is inappropriate, especially considering that impairments in driving relevant cognitive domains may not be distinct enough to justify the revocation of the driver licence. The guidelines for assessment of driving fitness do not generally exclude the possibility of participation on road traffic; yet 13,6% of MCI-patients don’t pass an on-road driving test^22^.

In modern societies mobility is essential regarding life quality and well-being. Consequently, in the interests of protecting patients and other road participants, the governmental revocation of driver licences must be critically weighed against values like preservation of self-confidence, freedom, self-responsibility and independence ^23^. Immobility often leads to social isolation, depression and a high level of stress for the family members caring for the patient^24-28^. That is why many older people are afraid of impending loss of their driver licence, and there is lack of knowledge concerning legal consequences neglecting their duty to precaution (“Vorsorgepflicht”, a German legal term describing ones duty to independently check up fulfilment of statutory requirements concerning driving ability). A possible starting point is a low-threshold, informal mobility counselling. In doing so licensed general practitioners, neurologists, psychiatrists etc. play a central role in this field, as they are often the first to be confronted with this topic and can sensitize the patient for it.

In clinical practice the issue of fitness to drive is associated with great uncertainties, both on practitioner’s and patient’s side^6,7^. Hence there is a distinct necessity of valid assessment instruments. Frequently brief-screenings such as the MMSE (Mini-Mental-State-Examination) are applied, which allow for a rough assessment of the cognitive status, although study results concerning the predictive value on driving safety are unsatisfying^29^. The results of neuropsychological tests, especially in domains like attention visuospatial perception, show moderate correlations with real on-road driving performance^30,31^. The observation of driving behaviour in an on road test, which is often considered a gold standard of investigation of the driving behaviour, cannot be provided usually due to its resource intensity.
Generally, single tests are not appropriate as indicators for driving performance, as they do not provide a comprehensive picture of the driving behaviour, have no or only a low predictive value and cannot achieve high sensitivity or specificity values^30,31^. Single tests merely provide indications of possible limitations of driving ability, but they cannot replace an on-road driving assessment^32-34^. Bennett & Hons (2016)^35^ stated that there is not an exclusive cognitive domain that reliably and validly represents fitness to drive^17^. It led to the conclusion that a comprehensive test battery consisting of test-combinations and multiple instruments, which survey different driving relevant cognitive functions, constitutes the best predictor for driving ability in elders with mild cognitive impairment^30,36,37^. Nonetheless the insufficient clarification of the validity criteria of these test-batteries has to be mentioned^38^, which should be accomplished by further instruments proximal and distal to the construct.
Furthermore there is a large gap between scientific evidence and clinical application, mainly impeded by the missing cut-off values of the majority of research results^29^. Only few studies eventually generated scores which allowed for a distinction between “fit to drive” and “unfit to drive”. Latest recommendations imply a classification accuracy of 80-90%^39^ to guarantee a consistently accurate prediction. In addition, in contrast to the previous dichotomization between safe and unsafe drivers, a trichotomization is suggested^29^ which should categorize drivers into “safe”, “uncertain” and “unsafe”, with the middle category entailing a more detailed assessment for accurate classification, e.g. with the aid of an on-road driving assessment. Developing this screening-tool highest importance should be ascribed to an efficient implementation in clinical practice. In other words, it should not be resource-intensive in terms of time and personnel, therefore suggesting a computer or tablet-based application.

The following research hypotheses can be deduced:

(1) We assume that mild cognitively impaired and healthy subjects will differ significantly regarding on-road driving performance.
(2) Among assessed cognitive domains we expect especially visuospatial performance, attention and executive functions to predict driving ability.
(3) We expect the newly developed screening-tool to differentiate with high diagnostic accuracy between safe and unsafe drivers. The accuracy is estimated at a minimum of 80% and should exceed the accuracy of single neuropsychological tests.
(4) Besides it is expected that driving competence will additionally be predicted by factors like age, driving experience in years and mileage per year.


**4. Study aims**The aim of the study is to develop a screening-tool with high predictive validity regarding driving ability of cognitively impaired subjects. Construct validity and criterion-based validity should be tested through a standardized on-road assessment as well as cognitive neuropsychological tests proximal and distal to the construct. The high practical relevance results from the necessity of a screening-tool for driving ability which is economically, validly and reliably applicable, tablet-based and thus mobile usable in medical care practice to support a low-threshold, qualitatively valuable and safe assessment of fitness to drive and counselling with respect to mobility; not least to meet legal requirements on the practitioner’s and patient’s side in this field.


**5. Outcomes**

**Primary outcomes**
Primary outcomes comprise performance in the neuropsychological assessment, especially in domains of attention, visuospatial abilities and executive functions.
Furthermore performance in the on-road assessment – at specified observational points - can be measured distinctively regarding errors made in categories like longitudinal control, lateral control and cognition. Every observational sequence is additionally rated on an 11-point rating scale.

**Secondary outcomes**
In addition sociodemographic, clinical and driving specific data will be analyzed with respect to their influence on the prediction of driving behavior.


**6. Study design**
The study is designed as a monocentric, non-randomized cross-sectional-study at the kbo-Inn-Salzach-Klinikum Wasserburg/Inn. Cut-off-scores in the MMSE of < 27 and ≥ 18 allocate the participants to the experimental group of subjects with mild cognitive impairment. Subjects with a MMSE-score ≥ 27 will be assigned to the healthy control group. We expect the recruitment period to last about 24 months. Each of these groups undergo the same assessment procedure. Blinding is only possible for the driving instructor.


**7. Study population**Women and men with an age ≥ 50 years, who possess a valid driver licence and who are active drivers, i.e. have been driving regularly the last three month before participation, and speak fluently German language can participate in the study. Regarding possible use of medication an exact documentation and clarification of potential adverse effects on on-road driving should be carried out in the context of anamnesis. Subjects who are under dose escalation or medication shifts will be excluded. The consent ability has to be checked, doubts about it also lead to exclusion. 40 subjects with mild to moderate cognitive impairment and 40 healthy control subjects will be assessed, parallelized regarding age, education, sex, driving experience and mileage per year.

*General exclusion criteria for all participants (based on annex 4, statutory order of driving licences)*

- Current severe dementia (MMSE < 18)
- Current severe psychiatric, neurological or internistic disease
- Visual impairment (visual acuity worse than 60%; visual field <140°, double-vision, hemi-neglect)
- No fluent German in speech and writing

*Specific criteria for the experimental groups*Healthy subjects (CG):

- ≥ 50 years
- German as first language respectively German-speaking since early childhood
- MMSE ≥27
- No record of severe psychiatric or neurological diseases
- Currently no internistic disease
- Currently no radio- or chemotherapy for cancer treatment

Cognitively impaired subjects (EG):

- ≥ 50 years
- First language German respectively German-speaking since early childhood
- MMSE ≥18 and <27
- Patients from the following ICD-10 diagnosis groups
  - Organic, including symptomatic disorders (F00-F09), N=20
  - Affective disorders (F30-F39), N=10
  - Schizophrenia, schizotypal or delusional disorders (F20-F29) / Neurotic, stress-related and somatoform disorders (F40-F48), N=10

For recruitment employees of the kbo-Inn-Salzach-Klinikum in Wasserburg/Inn will be addressed by mail and displays on the clinic campus as well as advertisement in relevant magazines and information-flyers. We request to make interested friends and other contacts aware of the study. Besides, patients will be informed about the study via the clinical departments for the diagnosis groups F00, F20 and F30 of the Inn-Salzach-Klinikum.

An estimation of the severity of possible psychiatric, neurological or internistic diseases will be made by the attending physician/psychologist of the particular department through anamnesis, information from the case history and medical reports. Assessing the healthy control group the principal investigator (psychologist) has to rely on the subjects’ truthful statements to make a classification.

**8. Study procedure**

Potential participants are initially informed about the study in written and verbally before given their written consent.

The assessments will take place at one term. Initially there is a briefing which contains the enlightenment (informed consent) and anamnesis as well as the allocation to experimental and control group by means of the MMSE-results. Subsequently the neuropsychological assessment is carried out which comprises the CFD test-battery (Cognitive Functions Dementia) and the Clock-Drawing-Test. The duration amounts to 120 minutes.

After a short recovery phase the on-road driving assessment is conducted. Finally a feed back is given on the results of neuropsychological assessment and the on-road driving. Moreover the participant is counselled regarding potential further therapeutic treatments.

| **Diagnostics**  Enlightenment  Consent  Anamnesis  Mini-Mental-State-Examination (MMSE)  Group allocation (by MMSE-Score)  Assessment of psychopathology   - Mini-SCL - PDQ   Assessment of driving competence (TQ-Drive)  Neuropsychological assessment   - CFD - Clock-Drawing-Test (CDT) - LAT (German adaption oft he JLO)   Duration: ca. 120 minutes | **On-road driving observation**  **& counselling**  On-road driving observation  Feedback oft he results   - Diagnostics - Driving behavior   Information leaflets  Duration: ca. 100 minutes |
| --- | --- |

Figure 1: Description of study procedure

*Enlightenment and anamnesis*a) Enlightenment and information brokerage
b) Informed consent
c) Anamnesis

*Deployed neuropsychological assessment tools*

| **Test** | **Mode** | **Duration** | **Annotation** |
| --- | --- | --- | --- |
| MMSE |  | 10 | CERAD-Plus Version |
| Mini-SCL, PDQ |  | 5 |  |
| Test-Set CFD | S1 | 60 | Standard mode plus shortened VISCO S3 mode |
| CDT |  | 3 |  |
| LAT | S1 | 10 |  |
| Total |  | 83 |  |

Table 1: Overview of the neuropsychological assessment

a) Mini-Mental-State-Examination (MMSE) – duration ca. 10 minutes
The Mini-Mental-State-Examination (Folstein et al., 1975)^40^ is a screening tool applicable in clinical practice to assess cognitive deficits. It is considered to be a reliable tool for initial examination of patients, e.g. in the context of dementia, as well as follow-up monitoring. The Mini-Mental-State-Examination is carried out as an interview with the patient. On the basis of 9 set of tasks central cognitive domains are checked (temporal and local orientation, memory retention, attention, language and speech comprehension, reading, writing, drawing and calculating). The MMSE tasks comprise both answering of questions and performing simple actions.

b) Assessment of subjectively perceived complaints and symptoms
The PDQ (Perceived Deficits Questionnaire; Fehnel et al., 2016)^41^ is utilized for the self-rating of cognitive deficits and allows for statements about the subjectively in everyday life perceived cognitive complaints of the patients and an estimation of its appearance and frequency. The reference period covers the last seven days.
The Mini-Symptom-Checklist (Mini-SCL; Franke, 2017)^42^ measures the subjectively perceived complaints using predefined physical and psychic symptoms over the last seven days.

c) Assessment of driving competence (TQ-Drive) – duration ca. 7 minutes
This computer based questionnaire assesses driving anamnestic information such as driving experience in terms of mileage per year, personal importance of the motor vehicle, driving difficulties and avoidance behavior etc. It provides a general impression of the individual driving performance and potential traffic-relevant risk factors.

d) Test-Set Cognitive Functions Dementia (CFD) – duration ca. 60 minutes
The test-set CFD measures neuropsychological domains relevant for neurocognitive disorders (according to DSM-5). By means of the CFD test-set cognitive performances in neuropsychological domains like attention, verbal long-term memory, executive functions, expressive speech and perceptual motor functions can be assessed. Table 2 lists all individual tests used in the study in order of display including duration of implementation.

|  | Dimension | Test | Subtest | Duration |
| --- | --- | --- | --- | --- |
| 1 | Semantic fluency | WIWO | S1 | 3 |
| 2 | Lexical fluency | WIWO | S3 | 4 |
| 3 | Learning ability | AWLT  (Subtest 1) | S1 | 7 |
| 4 | Alertness (intrinsic-visual) | WAFA | S2 | 2 |
| 5 | Brief delayed recall | AWLT  (Subtest 2) | S1 | 2 |
| 6 | Divided attention | WAFG | S3 | 9 |
| 7 | Processing speed | TMT | S1 | 1 |
| 8 | Cognitive flexibility | TMT | S1 | 1 |
| 9 | Working memory, spatial | CORSI | S7 | 8 |
| 10 | Long delayed recall and discrimination | AWLT  (Subtests  3 und 4) | S1 | 4 |
| 11 | Object naming | WOBT | S1 | 5 |
| 12 | Visuo-construction | VISCO | S3 | 7 |

Table 2: Dimensions and tests of the CFD test-set

The tests quoted in table 2 are presented in detail below.

On 1. and 2.: Vienna Verbal Fluency Test (WIWO)
Verbal Fluency describes a person’s ability to generate and reproduce words according to defined rules. There are two subdimensions of verbal fluency: semantic and lexical verbal fluency. Semantic verbal fluency describes a person’s ability to reproduce words of a specific category (e.g. first names) whereas lexical verbal fluency determines the ability to generate words beginning with specific letters.
Both subdimensions of verbal fluency are assessed in the CFD test set using the WIWO test. The respondent’s task is to name as many words as possible within two minutes that belong to a certain category (semantic verbal fluency: test form S1) or that start with a certain letter (lexical verbal fluency: test form S2). When the CFD test set is scored the main variable “verbal fluency” is calculated based on the number of correctly named words.

On 3., 5. and 10.: Auditory Word List Learning Test (AWLT):
Long-term memory describes the ability to retain information long-term, integrate it into one’s knowledge base and retrieve it when needed. Long-term memory contains all information that can be remembered for longer than a period of a few minutes. The process of learning is divided into the stages of encoding consolidation and recall.
Verbal long-term memory is assessed in the CFD test set with the AWLT test using test form S1. A list of 12 words is used as learning material in AWLT. The test implements a learning assessment method of four learning runs and short (5-minute break) and long (20-minute break) delayed free recall. As many words as possible must be freely reproduced immediately after each learning run and after the brief and long delays. The word list is not displayed again during the two recall phases. At the end, a list of 24 words is presented to the respondent: 12 words from the learning phase list and 12 new words intended as distractors. The respondent’s task is to say which of the 24 words were on the word list and which were not. The scoring of the CFD test set defines “learning total”, “short-term delayed recall”, “long-term delayed recall” and “recognition” as the main measurement variables.

On 4.: Perception and Attention Functions – Alertness (WAFA)
Alertness pertains to the core dimensions “Attention”. The construct of alertness encompasses both the state of ongoing general wakefulness and readiness to react (tonic alertness) and the ability to increase the level of attention quickly in response to a cue (phasic alertness). If stimuli are presented without a cue, the term “intrinsic alertness” is also used. Unlike phasic alertness, intrinsic alertness involves a self-generated increase in the level of alertness.
Alertness is measured in the CFD test set with the WAFA test using test form 2. The WAFA test measures reaction time in response to simple visual stimulus material (black circle on white background). The main variable that is reported when scoring the CFD test set is “Mean Reaction Time”.

On 6.: Perception and Attention Functions – Divided Attention (WAFG)
Divided attention pertains to the core dimension “Attention”. Divided attention is the ability to direct one’s attention toward several information channels simultaneously. For example, division of attention is required in “dual tasks” in which two information channels must be monitored simultaneously. If a relevant event occurs in one or both channels, the respondent must react as quickly as possible.
Divided attention is measured in the CFD test set with the WAFG test using test form 3. The respondent receives stimuli on one visual and one auditory channel. The task is to constantly monitor both channels to determine whether one of the target stimuli (square or high tone) changes twice in succession. The main variable that is reported when scoring the CFD test set is “Mean Reaction Time”.

On 7.: Trail-Making Test – Langensteinbach Version (TMT) Part A:
Processing speed is the basal ability to process simple stimuli quickly and with confidence without it being essential to involve higher cognitive, sensory or motor processes. The CFD test set assesses a fundamental measurement of processing speed using part A of the TMT test using test form S1. The task involves multiple circles containing the numbers from 1 to 25 that are presented simultaneously on the screen. These must be linked in ascending order as quickly as possible. The main variable that is reported when scoring the CFD test set is “Working Time Part A”.

On 8.: Trail-Making Test – Langensteinbach Version (TMT) Part B
Part B of the TMT assesses cognitive flexibility using test form S1. Cognitive flexibility is the ability to shift at will between different reference systems when this is required. The respondent’s task is to link circles containing the numbers 1 to 13 and the letters A to L alternately in ascending order. The main variable reported when the CFD test set is scored is the “Working Time in Part B”.

On 9.: CORSI Block-Tapping Test (CORSI)
Spatial working memory pertains to the core dimension “Executive Functions”. Working memory comprises the ability to process memory content in the mind in order to carry out tasks and achieve goals. Working memory can therefore be regarded as the ability to retain and process information that has been briefly presented and that is relevant to solving a task that is currently at hand.
(Spatial) working memory is assessed in the CFD test set with the CORSI test using test form S7. In this test nine blocks are shown on the screen. Each item involves a hand icon that moves about the screen, tapping on a certain number of blocks in a particular order. The respondent must tap the blocks in reverse order (backwards). The length of the sequences increases over the course of the test. It begins with a sequence of two taps and ends at the latest of a sequence of nine taps. The test terminates as soon as three successive sequences have been incorrectly tapped. The main variable reported when the CFD test set is scored is the variable “Immediate Block Span Backward”.

On 11.: Vienna Object Naming Test (WOBT)
Object naming pertains to the core dimension “Expressive Language”. Object naming describes the ability to recognize objects and to recall and reproduce the relevant names from one’s vocabulary. Declarative memory thus plays a role in test performance. Object naming is measured in the CFD test set with the WOBT test using test form S1. In the test, pictures depicting different objects are shown to the respondent and it is the respondent’s task to name these objects correctly. Should the respondent not be able to name the object, a lexical cue (the first letter of the target name) and after that a semantic cue (description of the target object without naming the target word) is given if needed. If none of these cues help the respondent to name the object, move on to the next item. The maim variable reported when the CFD test set is scored is the number of immediate correct namings (“Correct Naming Immediate”).

On 12.: Visuoconstruction Test (VISCO)
Visuoconstruction ability pertains to the core dimension “Perceptual Motor Functions”. Visuoconstruction refers to the ability of recognizing individual elements of shapes or objects and (re)constructing these shapes or objects by assembling the individual elements.
This ability is measured in the CFD test set with the VISCO test using test form S3. The test involves 16 tasks. For each task a shape made up of several equilateral triangles is shown as a target shape. Only the overall outline of the figure is visible, not the outline of the individual triangles themselves. The respondent’s task is to visualize the composition if the target figure and assemble it in an input field with the triangles that point both upwards and downwards. The respondents are given 60 seconds to solve each item. As soon as a person fails to complete the correct figure three times in succession, the test is automatically terminated. The main variable reported when the CFD test set is scored is the variable “Visuoconstruction Ability”.

e) Clock Drawing Test (CDT)
The Clock Drawing Test (Shulman, 1993)^43^ is a commonly used screening tool to assess visuoconstructive abilities for example during first diagnostic confirmation of dementia. The patient is asked to draw a clock face and enter the position of the clockhands for a certain time. The implementation takes about three minutes. It is possible to draw inferences about the extent of the dysfunction based on the deviations of depiction from “normal-performance” (segmentation of the clock face, type face of the digits, absence of the clockhands). In doing so 1 to 6 points are assigned depending on the deviation, whereas a score ≥ 3 can be classified as an indication of dysfunctions in this domain.

f) Line Adjustment Test (LAT)
Cerebral visuospatial processing are commonly differentiated into four categories: spatial-perceptual, spatial-cognitive, spatial-constructive and spatial-topografical performances. Spatial-perceptual performances are comparatively elementary. These include identification of the position of the subjective main space axes (vertical, horizontal), orientation (inclination), registration of the space within objects (length, size) and between objects (distances), recognition of positions and shapes, estimation of forms and also more specific performances like line halving and subjective straight orientation.
The LAT assesses basal spatial-perceptive abilities, especially the ability to estimate the inclination of lines and angles between lines. On one side of the screen two arrows are shown touching each other on the base in a certain angle. On the other side of the screen there is a clock-face. On that clock-face those two numbers have to be marked which are being pointed at by the arrows if those were situated in the middle of the clock-face. The main variable is the number of correctly handled tasks.

g) On-road driving assessment
The Wasserburg on-road driving assessment (WAFAB) is conducted by a certified driving instructor who is experienced assessing drivers with neurological or psychiatric disorders and a psychological technical assistant in a standardized driver instruction vehicle. It comprises a 50 minute drive on a predetermined route of approximately 50 kilometers length with determined observational spots and has been tested and established in clinical practice over the last ten years. The driving instructor is blinded against group allocation. As a global measure every observation sequence will be rated on an 11-point Fitness-to-Drive-Scale^44^. The scale consists of three verbal categories (normal, limited, citical) and within each of them three numeric subcategories (high, middle, low). In addition the driving errors will be rated by trained persons regarding total amount of errors, tactical errors with respect to longitudinal control (speed too high, time headway too low/tailgating), operational errors with respect to lateral control (lane departures/bad lane keeping, lateral distance to objects/vehicles too low) and cognitively based tactical errors (errors in changing/choosing lane, driving on impermissible lanes, securing behavior, communication, navigation errors) on determined observational spots; as an ancillary category critical situations (threats to other traffic participants, collisions) will be gathered.


**9. Benefit-risk analysis**At the time of assessment the participants are subjected to a test situation. This could be burdensome and provoke unpleasant feelings. However there is a detailed enlightenment about possible load factors. At each instant direct contact to psychologically trained staff is assured to guarantee quick announcement of complaints and an immediate reaction. Moreover, the study procedure can be cancelled at any time by the participants. The occurrence probability of further risks is estimated low. At any given moments participants can call for rest periods. In context of their participation, participants receive a free and professional assessment of their driving safety and are reimbursed with 25 Euro.
Establishing a new fitness-to-drive screening it is essential to know its predictive validity and accuracy. We consider the benefit-risk ratio to be fortunate because of the minor risks and major benefits of an economical and highly predictive fitness-to-drive screening for clinical daily routine.


**10. Biometry**

Examining the correlation between driving behavior and cognitive assessment in patients with neurological disorders studies indicate an average to large effect sizes for the TMT (part A and B) and visuospatial tests such as the Judgement of Line Orientation Test^16,17,22,45^. Therefore it can be assumed that TMT-L (part A and B) and LAT can predict driving behavior with an average effect size. The calculated sample size of N = 80 results as follows: Supposing a power (1 – β) of 80% and an alpha-level of α = .05 a sample size of N = 77 is necessary to detect an average effect calculating a multiple regression with three predictors (TMT-L part A, part B and LAT). In case of early dropouts we calculate a sample size of N=80 for recruitment.
Data will be analysed by means of multivariate analyses of variances followed by post-hoc-t-tests as well as regression analyses evaluating the predictive validity of the individual tests.
Premises of statistical calculations – such as normal distribution of sample or homogeneity of variance – shall be checked upfront.


**11. Data management**
All data collected in the context of the study exclusively serve research purposes, are kept in confidence and are anonymized irreversibly after elicitation plus are saved for an indefinite period. The results will be analysed after finalization of the study and will be published in anonymized manner which prevents inference to the participant’s person.


**12. Insurance**
The study is covered within the framework of the [employer's](https://www.dict.cc/?s=employer%27s) [liability](https://www.dict.cc/?s=liability) [insurance](https://www.dict.cc/?s=insurance) as well as the driving school’s insurance.


**13. Signature**


_______________________________________________________
Wasserburg/Inn, date

**14. References**

1. Anstey, K. J., Wood, J., Lord, S., & Walker, J. G. (2005). Cognitive, sensory and physical factors enabling driving safety in older adults. *Clinical psychology review*, *25*(1), 45-65.

2. McKhann, G. M., Knopman, D. S., Chertkow, H., Hyman, B. T., Jack, C. R., Kawas, C. H., ... & Mohs, R. C. (2011). The diagnosis of dementia due to Alzheimer’s disease: Recommendations from the National Institute on Aging-Alzheimer’s Association workgroups on diagnostic guidelines for Alzheimer's disease. *Alzheimer's & dementia: the journal of the Alzheimer's Association*, *7*(3), 263-269.

3. Smits, L. L., Pijnenburg, Y. A., van der Vlies, A. E., Koedam, E. L., Bouwman, F. H., Reuling, I. E., ... & van der Flier, W. M. (2015). Early onset APOE E4-negative Alzheimer’s disease patients show faster cognitive decline on non-memory domains. *European Neuropsychopharmacology*, *25*(7), 1010-1017.

4. Evans, L. (2004). *Traffic safety*.

5. McKnight, A. J., & McKnight, A. S. (1999). Multivariate analysis of age-related driver ability and performance deficits. *Accident Analysis & Prevention*, *31*(5), 445-454.

6. Jones, K., Rouse-Watson, S., Beveridge, A., Sims, J., & Schattner, P. (2012). Fitness to drive: GP perspectives of assessing older and functionally impaired patients. *Australian family physician*, *41*(4), 235.

7. Jang, R. W., Man-Son-Hing, M., Molnar, F. J., Hogan, D. B., Marshall, S. C., Auger, J., ... & Naglie, G. (2007). Family physicians’ attitudes and practices regarding assessments of medical fitness to drive in older persons. *Journal of general internal medicine*, *22*(4), 531-543.

8. Hedden, T., & Gabrieli, J. D. (2004). Insights into the ageing mind: a view from cognitive neuroscience. *Nature reviews neuroscience*, *5*(2), 87.

9. Golz, D., Huchler, S., Jörg, A., & Küst, J. (2004). Beurteilung der Fahreignung. *Zeitschrift für Neuropsychologie*, *15*(3), 157-167.

10. Falkenstein, M., & Sommer, S. M. (2008). Altersbegleitende Veränderungen kognitiver und neuronaler Prozesse mit Bedeutung für das Autofahren. *Prof. Dr.-Ing. Bernd H. Müller Forschungsstelle Mensch-Verkehr der Eugen-Otto-Butz-Stiftung*, 1885.

11. Lundberg, C., Johansson, K., Ball, K., Bjerre, B., Blomqvist, C., Braekhus, A., ... & Friedland, R. P. (1997). Dementia and driving: an attempt at consensus. *Alzheimer disease and associated disorders*, *11*(1), 28-37.

12. Anstey, K. J., & Wood, J. (2011). Chronological age and age-related cognitive deficits are associated with an increase in multiple types of driving errors in late life. *Neuropsychology*, *25*(5), 613.

13. Kroll, G., Kaiser, A., Krone, M., Mönning, M., Griese, H., Macek, C. E. E. A., & Hartje, W. (2003). Die praktische Fahrprobe im mittleren und höheren Lebensalter. *Zeitschrift für Neuropsychologie*, *14*(2), 81-87.

14. Deuschl, G., & Meier, W. et al. *S3-Leitlinie Demenzen. 2016.* In: Deutsche Gesellschaft für Neurologie, Hrsg. Leitlinien für Diagnostik und Therapie in der Neurologie. Online: [www.dgn.org/leitlinien](http://www.dgn.org/leitlinien) [22.11.2016]

15. Duchek, J. M., Carr, D. B., Hunt, L., Roe, C. M., Xiong, C., Shah, K., & Morris, J. C. (2003). Longitudinal driving performance in early‐stage dementia of the Alzheimer type. *Journal of the American Geriatrics Society*, *51*(10), 1342-1347.

16. Ott, B. R., Heindel, W. C., Papandonatos, G. D., Festa, E. K., Davis, J. D., Daiello, L. A., & Morris, J. C. (2008). A longitudinal study of drivers with Alzheimer disease. *Neurology*, *70*(14), 1171-1178.

17. Reger, M. A., Welsh, R. K., Watson, G., Cholerton, B., Baker, L. D., & Craft, S. (2004). The relationship between neuropsychological functioning and driving ability in dementia: a meta-analysis. *Neuropsychology*, *18*(1), 85.

18. Kessler, H., & Supprian, T. (2003). Zum Problem der Krankheitseinsicht bei Patienten mit Demenz vom Alzheimer-Typ.

19. Vogel, A., Stokholm, J., Gade, A., Andersen, B. B., Hejl, A. M., & Waldemar, G. (2004). Awareness of deficits in mild cognitive impairment and Alzheimer’s disease: Do MCI patients have impaired insight?. *Dementia and geriatric cognitive disorders*, *17*(3), 181-187.

20. Neumann-Zielke, L. (2004). Die Position Klinischer Neuropsychologen in der Rehabilitation von Kraftfahrern. *Zeitschrift für Neuropsychologie*, *15*(3), 189-207.

21. Albert, M. S., DeKosky, S. T., Dickson, D., Dubois, B., Feldman, H. H., Fox, N. C., ... & Snyder, P. J. (2011). The diagnosis of mild cognitive impairment due to Alzheimer’s disease: Recommendations from the National Institute on Aging-Alzheimer’s Association workgroups on diagnostic guidelines for Alzheimer's disease. *Alzheimer's & dementia: the journal of the Alzheimer's Association*, *7*(3), 270-279.

22. Hird, M. A., Egeto, P., Fischer, C. E., Naglie, G., & Schweizer, T. A. (2016). A systematic review and meta-analysis of on-road simulator and cognitive driving assessment in Alzheimer’s disease and mild cognitive impairment. *Journal of Alzheimer's disease*, *53*(2), 713-729.

23. Post, S. G. (2000). Key issues in the ethics of dementia care. *Neurologic clinics*, *18*(4), 1011-1022.

24. Fonda, S. J., Wallace, R. B., & Herzog, A. R. (2001). Changes in driving patterns and worsening depressive symptoms among older adults. *The Journals of Gerontology Series B: Psychological Sciences and Social Sciences*, *56*(6), S343-S351.

25. Marottoli, R. A., Leon, C. F. M., Glass, T. A., Williams, C. S., Cooney, L. M., Berkman, L. F., & Tinetti, M. E. (1997). Driving cessation and increased depressive symptoms: Prospective evidence from the New Haven EPESE. *Journal of the American Geriatrics Society*, *45*(2), 202-206.

26. Marottoli, R. A., de Leon, C. F. M., Glass, T. A., Williams, C. S., Cooney Jr, L. M., & Berkman, L. F. (2000). Consequences of driving cessation: decreased out-of-home activity levels. *The Journals of Gerontology Series B: Psychological Sciences and Social Sciences*, *55*(6), S334-S340.

27. Ragland, D. R., Satariano, W. A., & MacLeod, K. E. (2005). Driving cessation and increased depressive symptoms. *The Journals of Gerontology Series A: Biological Sciences and Medical Sciences*, *60*(3), 399-403.

28. Windsor, T. D., & Anstey, K. J. (2006). Interventions to reduce the adverse psychosocial impact of driving cessation on older adults. *Clinical interventions in aging*, *1*(3), 205.

29. Molnar, F. J., Patel, A., Marshall, S. C., Man‐Son‐Hing, M., & Wilson, K. G. (2006). Clinical Utility of Office‐Based Cognitive Predictors of Fitness to Drive in Persons with Dementia: A Systematic Review. *Journal of the American Geriatrics Society*, *54*(12), 1809-1824.

30. Dawson, J. D., Anderson, S. W., Uc, E. Y., Dastrup, E., & Rizzo, M. (2009). Predictors of driving safety in early Alzheimer disease. *Neurology*, *72*(6), 521-527.

31. Anderson, S. W., Aksan, N., Dawson, J. D., Uc, E. Y., Johnson, A. M., & Rizzo, M. (2012). Neuropsychological assessment of driving safety risk in older adults with and without neurologic disease. *Journal of clinical and experimental neuropsychology*, *34*(9), 895-905.

32. Brunnauer, A., Buschert, V., & Laux, G. (2014). Demenz und Autofahren. *Der Nervenarzt*, *85*(7), 811-815.

33. Iverson, D. J., Gronseth, G. S., Reger, M. A., Classen, S., Dubinsky, R. M., & Rizzo, M. (2010). Practice parameter update: Evaluation and management of driving risk in dementia report of the Quality Standards Subcommittee of the American Academy of Neurology. *Neurology*, *74*(16), 1316-1324.

34. Poschadel, S., Falkenstein, M., Pappachan, P., Poll, E., & Willmes von Hinckeldey, K. (2009). Testverfahren zur psychometrischen Leistungsprüfung der Fahreignung. *BERICHTE DER BUNDESANSTALT FUER STRASSENWESEN. UNTERREIHE MENSCH UND SICHERHEIT*, (203).

35. Bennett, J. M., Chekaluk, E., & Batchelor, J. (2016). Cognitive tests and determining fitness to drive in dementia: a systematic review. *Journal of the American Geriatrics Society*, *64*(9), 1904-1917.

36. Dawson, J. D., Uc, E. Y., Anderson, S. W., Johnson, A. M., & Rizzo, M. (2010). Neuropsychological predictors of driving errors in older adults. *Journal of the American Geriatrics Society*, *58*(6), 1090-1096.

37. Wood, J. M., Anstey, K. J., Kerr, G. K., Lacherez, P. F., & Lord, S. (2008). A multidomain approach for predicting older driver safety under in‐traffic road conditions. *Journal of the American Geriatrics Society*, *56*(6), 986-993.

38. Bowers, A. R., Anastasio, R. J., Sheldon, S. S., O’Connor, M. G., Hollis, A. M., Howe, P. D., & Horowitz, T. S. (2013). Can we improve clinical prediction of at-risk older drivers?. *Accident Analysis & Prevention*, *59*, 537-547.

39. Bedard, M., Weaver, B., Dārzin, P., & Porter, M. M. (2008). Predicting driving performance in older adults: we are not there yet!. *Traffic injury prevention*, *9*(4), 336-341.

40. Folstein, M. F., Folstein, S. E., & McHugh, P. R. (1975). “Mini-mental state”: a practical method for grading the cognitive state of patients for the clinician. *Journal of psychiatric research*, *12*(3), 189-198.

41. Franke, G. H. (2017). *Mini-Syptom-Checkliste.* Hogrefe.

42. Fehnel, S. E., Forsyth, B. H., DiBenedetti, B. D., Danchenko, N., François, C., & Brevig, T. (2016). Patient centered assessment of cognitive symptoms of depression*.* [*CNS Spect,*](https://www.ncbi.nlm.nih.gov/pubmed/24067243)*21(1)*, 43-52.

43. Shulman, K. I., Pushkar Gold, D., Cohen, C. A., & Zucchero, C. A. (1993). Clock‐drawing and dementia in the community: A longitudinal study. *International journal of geriatric psychiatry*, *8*(6), 487-496.

44. Neukum, A., & Krüger, H. P. (2003). Fahrerreaktionen bei Lenksystemstörungen–Untersuchungsmethodik und Bewertungskriterien. *VDI-Berichte*, *1791*, 297-318.

45. Grace, J., Amick, M. M., D'abreu, A., Festa, E. K., Heindel, W. C., & Ott, B. R. (2005). Neuropsychological deficits associated with driving performance in Parkinson's and Alzheimer's disease. *Journal of the International Neuropsychological Society*, *11*(6), 766-775.

46. Faul, F., Erdfelder, E., Lang, A. G., & Buchner, A. (2007). G* Power 3: A flexible statistical power analysis program for the social, behavioral, and biomedical sciences. *Behavior research methods*, *39*(2), 175-191.
